# Autistic and ADHD adolescents respond differently to upset in school: evidence from a new questionnaire co-designed from the neurodivergent point of view

**DOI:** 10.1007/s00787-026-03073-9

**Published:** 2026-06-01

**Authors:** Steve Lukito, Susie Chandler, Myrofora Kakoulidou, Kirsty Griffiths, Eloise Funnell, Anna Wyatt, Sylvan Baker, Daniel Stahl, Georgia Pavlopoulou, Edmund J. S. Sonuga-Barke

**Affiliations:** 1 Department of Child & Adolescent Psychiatry, Institute of Psychiatry, Psychology & Neuroscience, King’s College London, London, UK; 2 Group for Research in Relationships and Neurodiversity - GRRAND, Research in Clinical, Educational & Health Psychology, Division of Psychology & Language Sciences, Faculty of Brain Sciences, University College London, London , UK; 3 Research Department of Primary Care and Population Health, Institute of Epidemiology and Health Care, University College London, London, UK; 4 Royal Central School for Speech and Drama, London, UK; 5 Department of Biostatistics and Health Informatics, Institute of Psychiatry, Psychology & Neuroscience, King’s College London, London, UK; 6 Anna Freud National Centre for Children and Families, London, UK; 7 Department of Child and Adolescent Psychiatry, Aarhus University, Aarhus, Denmark; 8 Department of Psychology, Hong Kong University, Hong Kong, China

**Keywords:** Autism, Attention deficit/hyperactivity disorder, Emotional dysregulation, Adolescent mental health

## Abstract

Rates of depression are elevated in autistic and/or ADHD adolescents, but the reasons for this are unclear. We have previously shown that autistic and/or ADHD adolescents experience twice the school-related emotional burden of the non-ADHD/non-autistic control peers. Using a new self-report measure, the *My Emotional Responses in School Inventory* (MERSI), we now examine how autistic and/or ADHD students’ responses *after being upset*—often *pathologised* as “emotional dysregulation”—are associated with mental health. The 20-item MERSI was co-produced with neurodivergent young adults to measure adolescents’ responses after being made upset in school. It was administered to 732 secondary school students (ADHD [*n* = 100]; autism [*n* = 100]; ADHD+autism [*n* = 79]; non-ADHD/non-autistic control [*n* = 453]) alongside measures of depression, anxiety, emotion regulation difficulties, and emotional burden. The MERSI had three factors: (1*)* outward distress expressions, (2) inward distress expressions, and (3) solution-focused responses, and satisfactory reliability. Outward distress expressions were more likely for ADHD than non-ADHD, and for autistic than the control group. Autistic and/or ADHD students reported similarly higher inward distress expressions than controls. Solution-focused responses were less likely in ADHD. Inward distress expressions improved the prediction of depression and anxiety beyond emotional burden alone. Solution-focused responses were associated with lower anxiety in both ADHD groups relative to controls. Autistic and/or ADHD students respond differently after being upset at school than control peers. Our findings highlight the importance of monitoring students’ responses after being upset, especially in the cross-sectional prediction of anxiety and depression, to be further clarified through longitudinal data.

**Supplementary Information:**

The online version contains supplementary material available at https://doi.org/10.1007/s00787-026-03073-9.

## Introduction

Adolescence is a period characterised by opportunities for independence and psychological growth, but also by an increased likelihood of mental health challenges [1]. Such a likelihood is substantially elevated in adolescents with a diagnosis of autism [2] and/or attention-deficit/hyperactivity disorder (ADHD) [3]. By adulthood, approximately 50% of autistic people [4], and 30% of those with ADHD [5] have a full clinical depression and/or anxiety diagnosis, with the risk being greater among those with co-occurring ADHD and autism [6]. Understanding the mechanisms that explain why neurodivergent adolescents are at particular risk for mental health problems will be pivotal in finding better ways to prevent them.

The *Regulating Emotions – Strengthening Adolescent Resilience* (RE-STAR) programme explores how upsetting events and encounters, particularly in school, contribute to depression risk in ADHD and autism. To do this, RE-STAR employs a *deep-participatory* approach incorporating young neurodivergent students’ perspectives and experiences at the heart of its science [7–9]. Based on this collaboration, we hypothesised that depression risk affecting neurodivergent adolescents is mediated, at least in part, by a combination of increased levels of upsetting events and encounters experienced in non-neurodivergent-affirmative/inclusive environments and the more intense reactions these accumulating events evoke. We termed this *emotional burden*. Our first attempt to implement this approach led to the co-development, together with autistic and/or ADHD young adults [10], of the *My Emotions in School Inventory* [MESI; 11]. This measures the frequency of upsetting events, the intensity with which they are experienced, and their products summed across different upsetting events, which ultimately form an index of emotional burden. We found that students with autism and/or ADHD traits and/or diagnoses reported experiencing roughly twice the emotional burden of their control peers, and that emotional burden was associated cross-sectionally with levels of depression and anxiety, independently of emotion regulation deficits (ERDs) as measured by the Difficulties in Emotion Regulation Scale [12].

In this paper, we turn our focus to the responses young people make *after* being made extremely upset by provocations at school. Adolescents may respond to being upset in various ways, from seeking help, thinking of a solution to problems, to withdrawing and ruminating, which can either ease or intensify their distress and affect their long-term mental health [13, 14]. Emotional responding is a key part of emotion regulation [15], yet how individuals manage their experiences and expressions of emotions is often reported to be “deficient” or “dysregulated” in ADHD [16, 17] and autism [18–20]. In ADHD, emotional responding is typically interpreted as a lack of regulatory control, with intense anger or frustration manifesting as externalised behaviours such as verbal or behavioural outbursts [17, 21], with their own negative consequences. Conversely, in autism, emotional responses after being upset can include internalised behaviours such as ‘shut down’ or withdrawal, especially in response to overwhelm [22, 23] rapidly escalating negative affect or ‘meltdowns’ [24], or perseveration and rumination [25]. Adaptive coping strategies [15, 26] have been shown to be reduced in autistic adolescents [27, 28] and those with ADHD [29, 30] and this in turn has been linked to long-term mental health problems [31].

These ways of understanding or describing these experiences, however, are fundamentally grounded in neurotypical perspectives rather than lived neurodivergent experiences, as evident from the measures used to assess them. Existing self-report measures of emotional responding include the Emotional Behaviour Scale [32], Youth Self-Report [33], the Affective Reactivity Index [34], the Emotional Reactivity Scale [35], and the Emotion Dysregulation Inventory [36]. Except for the latter, these have not been developed for or validated with autistic and/or ADHD young people, and none relates to the young people’s responses to the context. Neurodivergent young people’s emotional responses are instead seen through a neurotypical lens based on whether or not they meet neuro-normative standards. Differential responses are thus viewed as reflecting deficits within the person, for instance in the ability to control or regulate emotional impulses [17], or to identify, understand/describe emotion, also known as alexithymia [37, 38], rather than stemming from high levels of emotional burden that are associated with school-related upsetting events such as peer problems or bullying [39, 40], conflicts with teachers [41], or non-social factors such as academic pressures or sensory triggers [42].

Co-produced with a panel of neurodivergent youth researchers alongside the MESI, the *My Emotional Responses in School Inventory* (MERSI) offers a novel way to measure emotional responding that captures the range of experiences in the student population. It is designed for use with *all* secondary school students, while also sensitively incorporating the experiences of those who are neurodivergent. It adopts a more neutral framing of the responses while highlighting the role of environmental provocations in school.

Understanding differential patterns in emotional responding informs the creation of supportive environments that promote the development of agency and resilience for longer-term positive mental health. Therefore, in this study, we used MERSI data from a large sample of adolescents, enriched for cases of autism and ADHD, to examine the following research questions: (1) *Are responses after being upset*,* following environmental provocation(s)*,* different among ADHD/autistic adolescents and their non-ADHD/non-autistic control peers?* We expected neurodivergent group differences relative to the control peers in the MERSI domains, reflecting their alignment with constructs such as externalising behaviour, which would be scored higher by the ADHD group; internalising behaviour, which would be scored higher by the autistic group; and coping, which would be scored lower in all neurodivergent groups. (2) *How does including these responses*,* alongside emotional burden*,* influence our cross-sectional ‘prediction’ of mental health problems (e.g.*,* depression or anxiety)?* We expected that this would improve the prediction of depression or anxiety in secondary school students. (3) *Are there differences in the association between these responses after being upset and mental health problems across groups?* We had no strong hypotheses for this question, but anticipated different association patterns across groups consistent with their potentially differing responses after being upset.

## Methods

### Participants

Adolescents aged 11–16 years (*n* = 746) were recruited through local NHS clinics, schools, and UK autism and ADHD charities [11]. Two hundred and thirteen had a verified parent-report of a diagnosis of either ADHD (*n* = 89), autism (*n* = 66), or both (*n* = 58). Participants were included if they attended a mainstream secondary school and had sufficient use of the English language to complete the questionnaire measures. They were assigned to ADHD, autism, or combined (i.e., ADHD+autism) group based on a parent-reported formal diagnosis of autism and/or ADHD, or a score above the clinical cut-off on screening measures [43, 44]. Those scoring below cut-offs and without an autism/ADHD diagnosis were assigned to the non-autistic/ADHD group, hereafter referred to as the control group.

## Measures

### Demographics

Parent/carers provided information about their child’s sex, age, diagnosis, and ethnicity.

## Responses after being upset in school

Adolescent participants completed the *My Emotional Response in School Inventory* (MERSI), alongside the MESI [11]. It asks, ‘What would you do if you got extremely upset at school? Items such as *‘get into a fight’*,* or ‘withdraw into yourself*,* shut everything out’* (See Supplementary Materials, Table S1) are rated on a likelihood scale (0 = *very unlikely*, 1 = *unlikely*, 2 = *possibly*, 3 = *likely*, 4 = *very likely*). The 24-item MERSI prototype was formulated from accounts of 57 neurodivergent secondary school students [see 7, 8 for details] and was piloted with 50 ADHD and/or autistic adolescents (mean age = 13.4 years, 44% female) [see 11]. Minor wording revisions were made based on their feedback before this investigation.

## ADHD traits

Parents completed the 26-item *SNAP-IV* [44] as a measure of their child’s ADHD traits. Recommended clinical cut-offs for inattention (1.78), hyperactivity (1.44), and combined (1.67) symptoms [45] were applied.

### Autism traits

Parents completed the 39-item *Social Communication Questionnaire* SCQ; (43) as a measure of their child’s autism traits. The recommended total score cut-off of 15 was applied [43].

## Emotional regulation deficits (ERD)

Young people rated 18-item Difficulties in the Emotion Regulation Scale (short form) [DERS; 12] statements, e.g., ‘When I’m upset, I become out of control’ about themselves (1 = *almost never*, 2 = *sometimes*, 3 = *about half the time*, 4 = *most of the time*, 5 = *almost always*). Higher total scores indicated greater ERD.

## Emotional burden (EB)

School-induced EB was measured using the self-report MESI. Young people rated upsetting school events such as: *‘Finding out your peers have been talking about you behind your back’*, or *‘School staff don’t listen to you or challenge what you say’*, on frequency (0 = *never*, 1 = *rarely*, 2 = *sometimes*, 3 = *often*, 4 = *frequently*) and degree of upset (0 = *not at all*, 1 = *a little*, 2 = *somewhat*, 3 = *a lot*, to 4 = *extremely*). Frequency and upset ratings were multiplied, then summed to generate the EB index [11], which is higher with increased emotional burden.

### Depression and anxiety symptoms

The *Patient Health Questionnaire* PHQ-9; [46] and the *Generalized Anxiety Disorder Scale* GAD; [47] were completed by young people to measure depression and anxiety symptoms, respectively. The original PHQ-9 includes nine items (e.g., ‘Feeling down, depressed or hopeless’); however, the ninth item on self-harm was removed for ethical reasons (PHQ-8 Cronbach’s alpha = 0.87). The GAD-7 includes seven items (e.g., ‘Feeling nervous, anxious, or on edge’). In both measures, these were rated in the last two weeks (0 = *not at all*, 1 = *several days*, 2 = *more than half the days*, 3 = *nearly every day*). Higher total scores indicated greater mental health problems.

### Alexithymia

Young people completed the Toronto Alexithymia Scale TAS; (48) to assess difficulties in identifying and describing emotions, which was included as a confounder in analyses of responses after being upset and mental health problems. They rated 20 items (e.g., ‘I find it difficult to say how I feel inside’) as 0 = *not true*, 1 = *a bit true*, and 2 = *true*. Higher scores indicated higher levels of alexithymia.

### Procedure

The study was approved by the NHS Health and Social Care Research Ethics Committee (HSC REC A; Ref 23/NI/230047). Eligible families were emailed information about the study and a link to the online consent form. On completion of parental consent and young person consent/assent, parents and young people completed the questionnaires online on a Qualtrics^XM^ platform (Provo, UT). A subsample of young people repeated the MERSI after two weeks for test-retest reliability examination.

### Sample size estimations

The sample exceeds the minimum sample size of *n* = 240 estimated for the exploratory factor analysis (EFA) for the prototype 24-item MERSI (Full consideration in [11]), by over threefold. The study had 0.90 power to detect small effect sizes for group differences, correlations, and regression predictors. The less well-powered subgroup analyses are exploratory. The test-retest reliability examination used a sample size of *n* = 52, allowing for a 10% drop off, for detecting absolute test-retest agreement by intraclass correlation (ICC) = 0.70 with a 95% confidence interval around the reliability of 15% [49].

### Statistical analysis

Statistical analyses were completed in R 4.1.1 on the RStudio 2023.12.1 using packages *Psych* [50], *Lavaan* [51], *MASS* [52], and *IRR* [53] and *boot* [54].

Preliminary item analysis followed a set of pre-specified criteria to identify items considered for removal (Supplementary Material, S1). Participants with missing MERSI scores were excluded from the analyses if they were fewer than 5% of the overall sample [55]. Group differences in participant characteristics were examined. *Post-hoc* pairwise comparisons were performed with Tukey correction.

MERSI structure was investigated using exploratory factor analysis (EFA) with ordinary least squares, applying *Oblimin* rotation for correlated factors. Scree plots [56] and parallel analyses [57] helped identify optimal factor number. Items were considered for removal if they: loaded on a factor with fewer than five indicators, unless their loadings were > 0.7 [58]; loaded with a factor loading < 0.4; or cross-loaded in more than one factor with a secondary loading of ≥ 0.3, or with a loading difference < 0.2 [59]. Model selection was aided by goodness-of-fit criteria, including likelihood ratio chi-square, Tucker-Lewis Indices (TLI; good fit ≥ 0.95 [60]), and the Bayesian information criterion (BIC) [61]. The model’s robustness was further examined using the bias-corrected bootstrap for skewed responses [62].

Internal consistency was indexed using Cronbach’s α and item-total correlation per domain. Further item removal was considered if α increased when an item was dropped or if item-total correlation was < 0.3 [59]. ICC examined agreement between MERSI latent factor and domain scores: in the overall sample, and in participant groups (ADHD, autism, combined, and control), and subgroups by sex (male, female), or ethnicity (recoded into white vs. minoritised ethnic groups, since groups other than the white ethnic group were too sparse for separate analysis). Two-week test-retest reliability of MERSI domain scores was examined using ICC with absolute agreement [59].

As an additional robustness examination, we assessed the measurement invariance of the final MERSI domains using a series of multi-group confirmatory factor analyses (MGCFA) across groups (ADHD, autism, combined, control), sex (male, female), and ethnicity (white, minoritised ethnic groups) (Supplementary Material S1). We tested configural, metric, and scalar invariance, although scalar invariance was not expected for this measure, as attitudinal data often exhibit mean group differences across groups. Invariance was evaluated using changes in fit indices (ΔCFI ≤ 0.005, ΔRMSEA ≤ 0.010), following recommended thresholds for unequal group sizes below *n* = 300 [63].

Before conducting analyses to answer the research questions, we examined bivariate pairwise correlation between MERSI and DERS domain scores, using Pearson’s *r* (Supplementary Material S1). We also examined the item score, and we tested whether subgroups of participants with or without formal diagnosis status differed in the MERSI scores by conducting a full-factorial 2(formal diagnosis status) × 3(group [ADHD, autism, combined]) analysis of variance (ANOVA) (Supplementary Material S1).

Then, to examine whether responses after being upset differ between the neurodivergent and control participants, we conducted a series of one-way ANOVAs with each domain of MERSI responses as dependent variables and group (ADHD, autism, combined, controls) as an independent variable. We conducted additional tests adjusting for age, sex, and ethnicity, given the differences in these variables across groups. Furthermore, to test for violation of the homogeneity of association assumption, we also added interaction terms between these variables and the participant group. Partial eta squared (*η*_p_*²*) was reported as the effect size for the ANOVA F-statistics, while adjusted Cohen’s *d* was used as the effect size for the pairwise comparisons. *Post hoc* pairwise comparisons of estimated marginal means (EMMs) were conducted using Tukey adjustment (Further details in Supplementary Material S1).

To investigate whether these responses improved the cross-sectional prediction of depression or anxiety alongside emotional burden (EB), we first examined the associations between each MERSI domain score and depression or anxiety using simple linear regressions in the overall sample. We then added these responses to a model in which EB predicted mental health and assessed changes in the adjusted R². The associations were further examined while controlling for confounders (i.e., age, sex, ethnicity, and alexithymia) as well as ERD, to assess the independent contributions of EB and responses after being upset from emotional dysregulation, as shown in our previous study [11]. Standardised beta (*b*) was used as an effect size measure in these regression analyses.

Finally, to explore differences in associations between responses after being upset and mental health across groups, we conducted a series of multiple regression analyses, entering group, MERSI domain scores, and their interaction term to predict depression or anxiety. We also conducted this analysis, while adjusting for the confounders (i.e., age, sex, ethnicity, and alexithymia).

## Results

### Preliminary analyses

Most MERSI items met the inclusion criteria for the initial item analysis (Supplementary Material S2, and Figure S1), but there was poor two-week item-level agreement for items: *‘Do something you find comforting’*, *‘Leave the situation to find your own space’*, and *‘Distract yourself by thinking about positive things’* (ICCs = 0.1–0.4) (Table S2). Fourteen participants (1.9%) with missing MERSI items were excluded, a level considered ‘inconsequential’ in terms of bias [55], leaving data from 732 participants for subsequent analyses (ADHD, *n* = 100; autism, *n* = 100; combined, *n* = 79; controls, *n* = 453]; mean age 13.3, SD 1.32 years; 36.1% female, 52.4% white). The groups differed in age, sex, and ethnicity, but had similar levels of EB, ERD, depression, and anxiety (Table 1).

**Table 1:** Participant characteristics

Variables	ADHD (*n* = 100)		Autism (*n* = 100)		Combined (*n* = 79)		Control (*n* = 453)			Group Statistics				Post-hoc
	M/*n*	SD/%	M/*n*	SD/%	M/*n*	SD/%	M/*n*	SD/%	X^2^/F		df	*P*		
Age	13.1	1.27	13.6	1.49	13.1	1.44	13.3	1.25	3.09		(3,731)	0.03	Autism> ADHD	
Sex (female)	29	29	56	56	31	39.2	148	32.7	7.48		(3,730)	< 0.001	Autism> ADHD, Control	
Ethnicity														
Asian	1	1	4	4	3	3.8	125	27.7	103.4		9	< 0.001	--	
Black	7	7	18	18	5	6.3	75	16.6						
Mixed	13	13	13	13	14	17.7	51	11.3						
White	77	77	64	64	56	70.9	186	41.2						
Autism trait														
SCQ total score	11.3	5.81	17.3	5.22	19.4	6.4	8.32	5.31	140.6		(3,728)	< 0.001	Combined> ADHD, Control, Autism; Autism> ADHD, Control; ADHD> Control	
ADHD trait														
SNAP total score	2.18	0.51	1.37	0.58	2.18	0.56	0.576	0.53	393.8		(3,731)	< 0.001	Combined> Autism, Control; ADHD> Autism, Control; Autism> Control	
EB														
MESI EBI	124.7	66.7	127.1	69.1	146.6	78.1	67.1	52.8	67.7		(3,723)	< 0.001	Combined, ADHD, Autism> Control	
ERD														
DERS total score	53.0	14.1	52.0	13.6	57.1	15.1	41.0	14.1	47.7		(3,705)	< 0.001	Combined, ADHD, Autism> Control	
DERS awareness	9.83	2.64	9.62	3.15	10.8	2.73	8.88	3.15	10.9		(3,715)	< 0.001	Combined, ADHD> Control	
DERS clarity	7.76	3.09	8.13	3.25	8.80	3.56	6.10	3.06	26.6		(3,719)	< 0.001	Combined, ADHD, Autism> Control	
DERS goal	11.0	3.17	11.0	3.42	11.6	3.50	8.17	3.69	40.4		(3,717)	< 0.001	Combined, ADHD, Autism> Control	
DERS impulse control	9.38	3.92	7.85	3.55	9.96	4.01	5.84	3.27	52.0		(3,717)	< 0.001	Combined, ADHD, Autism> Control; Combined> Autism	
DERS nonacceptance	7.51	3.64	7.27	3.63	7.31	3.79	5.99	3.23	9.27		(3,715)	< 0.001	Combined, ADHD, Autism> Control	
DERS strategies	7.60	3.36	8.02	3.19	8.32	3.63	5.91	3.19	22.8		(3,715)	< 0.001	Combined, ADHD, Autism> Control	
Depression														
PHQ-8 total score	8.80	5.24	9.32	5.54	9.56	6.23	5.48	5.46	26.2		(3,726)	< 0.001	Combined, ADHD, Autism> Control	
Anxiety														
GAD-7 total score	8.51	5.39	9.35	5.59	9.46	6.27	5.08	5.14	32.5		(3,727)	< 0.001	Combined, ADHD, Autism> Control	
Alexithymia														
TAS total score	20.2	6.93	20.2	7.18	22.4	7.35	15.1	7.12	38.1		(3,721)	< 0.001	Combined, ADHD, Autism> Control	
DDF subscale	5.46	2.75	5.88	2.60	6.21	2.88	4.15	2.82	21.4		(3,721)	< 0.001	Combined, ADHD, Autism> Control	
DIF subscale	6.66	3.77	7.06	4.00	7.52	4.06	3.88	3.65	41.0		(3,718)	< 0.001	Combined, ADHD, Autism> Control	
EOT subscale	8.05	2.58	7.21	2.85	8.61	2.46	7.09	2.51	10.4		(3,715)	< 0.001	Combined, ADHD, Autism> Control	

Characteristics of the included participants are described across groups, with group difference statistics presented. Abbreviations. M= mean, SD= standard deviation, n=participant number, SNAP = Swanson, Nolan and Pelham Rating Scale, SCQ=Social Communication Questionnaire, EB=emotional burden, MESI EBI = My Emotions in School EB Index, ERD=emotional regulation deficits, DERS = Difficulties in Emotion Regulation Scale, PHQ-8 = Patient Health Questionnaire, GAD-7 = Generalised Anxiety Disorder Questionnaire, TAS = Toronto Alexithymia Scale, DDF = difficulty describing feelings, DIF = difficulty identifying feelings, EOT = external-oriented thinking

### Exploratory factor analysis (EFA)

The scree and parallel plots suggested a three-factor structure (Supplementary Material, Figure S2). Initial EFA resulted in three factors with up to eight indicator items each. Three items were removed due to low- or cross-factor loadings (Supplementary Material, Table S3), and one item was removed because, on reflection, it was perceived as relating to a longer time response than the others (Supplementary Material S2).

The final 20-item MERSI contained three factors, with item-factor loadings *B*s = 0.48–0.76, item communalities *h*2 = 0.23–0.56 (Supplementary Material, Table S4), and an acceptable fit (Likelihood Chi2 = 782, *p* <.001; TLI = 0.814; RMSEA = 0.082, 90% CI [0.076, 0.087]; BIC = −95.7; SRMR = 0.05) explaining 42% of variance in the data. The factors were labelled: (1) ‘*Outward Distress Expressions (ODE)’*, (2) ‘*Inward Distress Expressions’ (IDE)*, and (3) *Solution-Focused Responses (SFR)*. Factors 1 and 2 correlated moderately and positively (*r* = 0.35). Factors 1 and 3 (*r* = −0.19) and Factors 2 and 3 (*r* = −0.13) correlated weakly and negatively (Table S4).

### Internal consistency and reliability

Internal consistency of the 20-item MERSI was good (Cronbach’s α = 0.83). Sequential removals of items mostly reduced the *α* (Table S4), with a few acceptable exceptions: *‘confront the person/situation’*, *‘look for explanations for why the situation has occurred’*, and *‘use strategies you’ve “learnt” to manage your feelings’*. Within the domains, internal consistency was ‘acceptable’ to ‘good’ (Cronbach’s α_ODE_ = 0.85; α_IDE_ = 0.81; α_SFR_ = 0.78), and item-average correlation suggested no redundancies or uncorrelations (*r* =.30–0.70). Item-total correlations were above 0.40 (*r*_ODE_ = 0.46–0.68; *r*_IDE_ = 0.47–0.64; *r*_SFR_ = 0.42–0.61; Table S4). Thus, no further items were removed.

Latent-factor and total domain scores had an excellent agreement overall (i.e., ICC_ODE_=0.99, ICC_IDE_= 0.99, and ICC_SFR_=0.99) and in subgroups defined by neurodivergence traits, sex, and ethnicity (ICCs = 0.98–0.99). Test-retest reliability assessment, conducted with 46 participants (9 ADHD, 13 autism, 9 combined, and 15 control; age = 13.4 years, female = 20 [43%], mostly white [82%]), was satisfactory (ICC_ODE_=0.67; ICC_IDE_=0.70; ICC_SFR_=0.69). The MGCFA investigations indicated suboptimal configural, metric, and scalar fits but with acceptable metric invariance across different subgroups (ΔCFIs ≤ 0.005, ΔRMSEA≤ 0.003), whether it was by neurodivergent traits (ADHD, autism, combined, and control), sex, or ethnicity (Table S7).

### Correlation between MERSI and DERS domain scores

The MERSI and DERS domain scores were correlated in the expected direction with effect sizes ranging from small to large (Supplementary Materials S2, Table S7).

### Response items and domain scores across groups

**Fig. 1:**
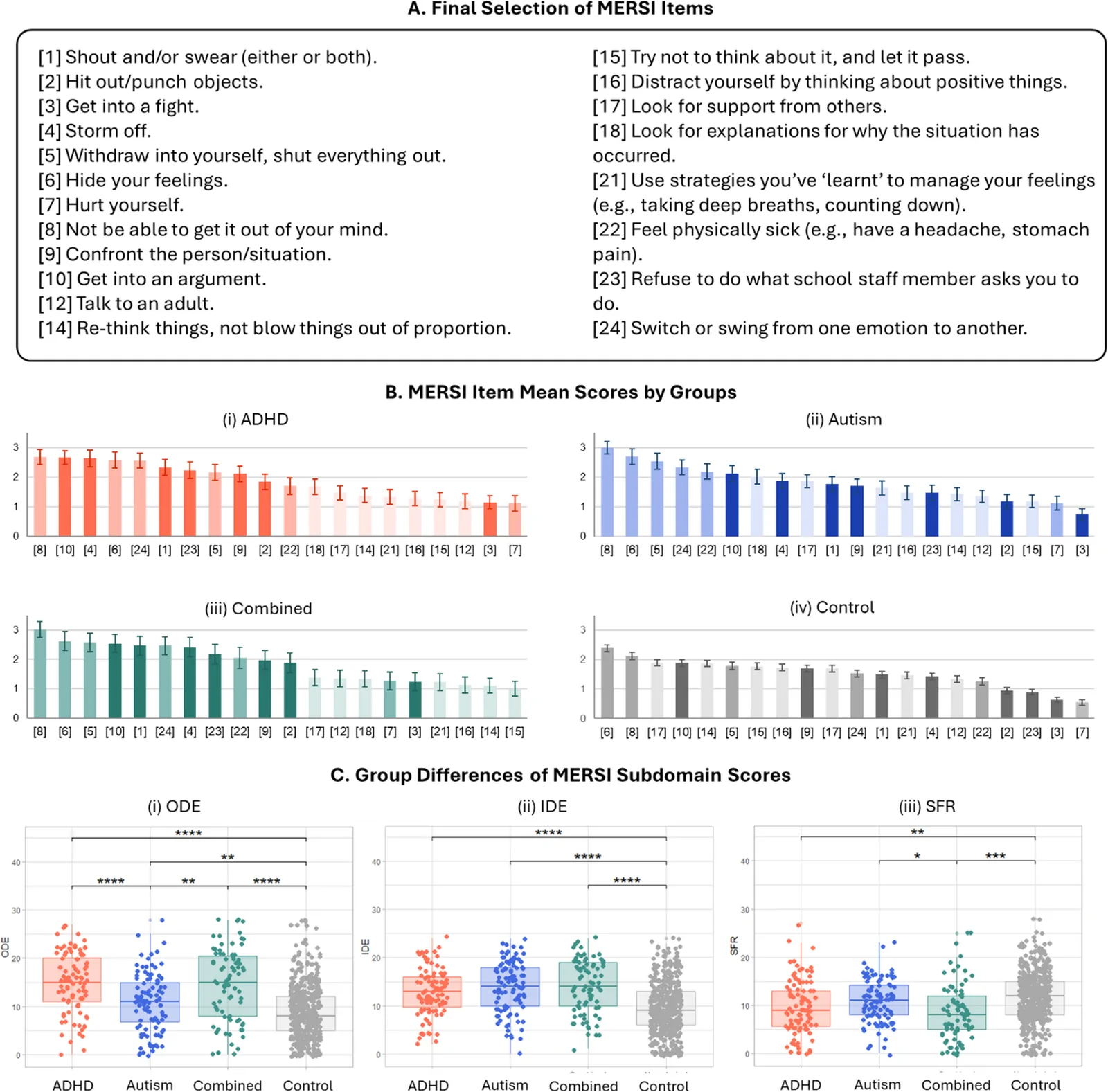
MERSI Item and Scores by Groups. Figure 1 shows (**A**) items included in the final MERSI—items 11,13,19,20 are excluded from the final scale; (**B**) MERSI item mean scores ranked from the highest to the lowest within each group (numbers on the x-axis indicate the item number in the scale as listed in (A))—items with darker hue reflects outward distress expression (ODE), middle hue reflects inward distress expression (IDE), and lighter hue reflects solution-focused responses; (**C**) Comparisons of MERSI domain scores across groups. Full reports on mean, SD, and rank of the individual MERSI items by groups are in Supplementary Table S6

Figure 1 presents the final MERSI items, and scores by group. Mean item and domain scores by group, with items ordered according to their mean ranks in the overall sample, are presented in Supplementary Material, Table S4. The highest-ranked items were ‘*hide your feelings’* and ‘*not be able to get it out of your mind*’, which are inward distress expressions. The highest-ranked outward distress expressions were ‘*get into an argument*’ and ‘*confront the person*’. In general, solution-focused responses ranked lower than those conveying distress expressions. The highest-ranked response of this kind was ‘*look for explanation’*.

In ADHD and the combined group, mean item scores (Fig. 1B) showed a mixed likelihood of inward/outward distress expression among the top-ranked items. Conversely, the five top-most responses of the autism-only group were *all* inward distress expressions. Solution-focused response items were among the lowest-ranked in both groups with ADHD.

Neither the interaction between formal diagnosis status and three neurodivergent groups (i.e., very small effect *η*_p_^2^s = 0.00001–0.002), nor the effect of formal diagnosis (very small effect *η*_p_^2^s = 0.001–0.005) was significant for all response types, with small and non-significant differences (*d*s = 0.03–0.36, *p*s = 0.16–091) between groups with/without diagnosis, supporting the decision to collapse subgroups with/without formal diagnosis within each group for the remaining analyses (Supplementary Materials S2, Table S8).

ANOVAs showed a main effect of Group for *all* domain scores (*F*_ODE_[3, 724] = 40.7, *η*^2^ = 0.14; *F*_IDE_[3, 724]= 31.3, *η*^2^ = 0.11; *F*_SFR_[3, 724] = 10.3, *η*^2^ = 0.04; all *p*s<0.0001) (Supplementary Material Table S5, Fig. 1C). All three neurodivergent groups expressed a higher likelihood of outward (ADHD: *ΔEMM =* 6.07, Cohen’s *d* = 1.00, *p*<.001; autism: *ΔEMM =* 1.97, *d* = 0.32, *p*=.018; combined: *ΔEMM =* 5.76, *d* = 0.94, *p*<.001) and inward distress expressions (ADHD: *ΔEMM =* 3.22, *d* = 0.60; autism: *ΔEMM =* 4.28, *d* = 0.79; combined: *ΔEMM =* 4.35, *d* = 0.81, all *p*s<0.001) than their control peers (Fig. 1C). Inward distress expressions did not differ among the neurodivergent groups (*d*s = 0.01–0.21; *p*s > 0.5). Outward distress expressions in the ADHD and combined groups, equivalent to each other, were higher than the autistic-only group (ADHD: *ΔEMM =* 4.10, *d* = 0.67; combined: *ΔEMM =* 3.77, *d* = 0.62, both *p*s<0.001). In line with the item-level findings, solution-focused responses were lower in both ADHD groups, relative to the control group (ADHD: *ΔEMM=-*2.16, *d*=−0.39, *p*=.002; combined: *ΔEMM=-*3.20, *d*=−0.58, *p*<.001), and in the combined group relative to the autistic-only group (*ΔEMM=-*2.44, *d*=−0.44, *p*=.017) (Fig. 1C).

Findings remained after adjusting for the group differences in age, sex, and ethnicity (Supplementary Materials S2, Table S10). Among these adjusted models, sex and ethnicity were associated with inward expression of distress (sex: *F*[3, 724] = 92.8, *p*<.001, *η*_p_^2^ = 0.10; ethnicity: *F*[3, 724] = 4.67, *p* =.03, *η*_p_^2^ = 0.005), and no other significant associations were found. Follow-up comparisons revealed that inward expression of distress was higher in females than males (*ΔEMM =* 3.72, *d* = 0.74, *p*<.001), and higher in white than minoritised ethnic groups (*ΔEMM =* 0.86, *d* = 0.17, *p*=.031). No significant interaction was found between participant group with age, sex, or ethnicity (Supplementary Table S10), suggesting that these variables did not modify the pattern of differences between groups.

### Relations between responses after being upset with mental health problems

**Fig. 2:**
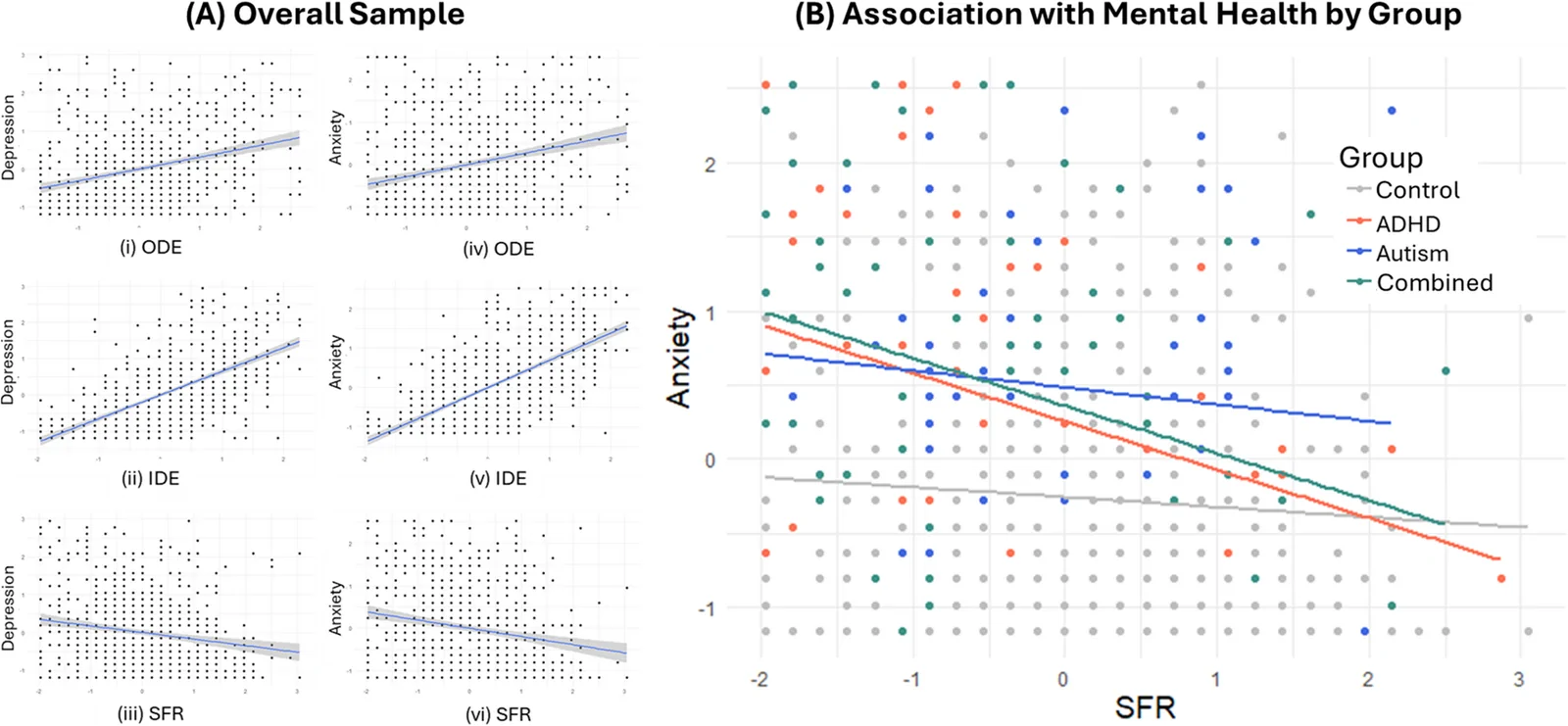
Associations between Domain Scores of MERSI, Depression, and Anxiety. (**A**) Associations in the overall sample between depression and MERSI domain scores of (i) outward distress expressions (ODE), (ii) inward distress expressions (IDE), and (iii) solution-focused responses (SFR), and between anxiety and the respective constructs (iv-vi); (**B**) The association between the MERSI SFR and anxiety in the ADHD and the Combined groups were significantly more negative compared to those in the autism-only and the control groups

Simple linear regressions with the overall sample found a significant positive association between both outward and inward distress expressions with depression (*b*_ODE_=0.31, 95%CI [0.24,0.38]; *b*_IDE_=0.65, 95%CI [0.60,0.71], *p*s<0.0001), and anxiety (*b*_ODE_=0.28, 95%CI [0.21,0.35]; *b*_IDE_=0.69, 95%CI [0.64,0.75], *p*s<0.0001). Conversely, solution-focused responses were negatively associated with mental health problems (depression: *b*_SFR_=−0.17, 95%CI [−0.24,−0.10]; anxiety: *b*_SFR_=−0.19, 95%CI [−0.26,−0.12], *p*s<0.0001) (Fig. 2 A).

Adding the responses alongside EB improved the explanatory model for predicting depression or anxiety by significantly increasing the *R*^*2*^ (depression: Δ*R*^*2*^ = 0.11, *F*[3, 714] = 52.4; anxiety: Δ*R*^*2*^ = 0.13, *F*[3, 715] = 70.8; *p*s<0.0001). In these predictive models, inward distress expressions was the strongest additional predictor of depression or anxiety among the responses (depression: *b*_IDE_=0.45, 95%CI [0.38,0.52]; anxiety: *b*_IDE_=0.48, 95%CI [0.42,0.53]; *p*s<0.0001), comparable in magnitude with the association between EB and mental health outcomes (depression: *b*_EB_=0.31, 95%CI [0.23,0.38]; anxiety: *b*_EB_=0.36, 95%CI [0.29,0.43], *p*s<0.0001). Outward distress expressions and solution-focused responses negatively predicted anxiety only, at a considerably lower strength (*b*_ODE_=−0.08, 95%CI [−0.13,−0.02], *p*=.006; *b*_SFR_=−0.06, 95%CI [−0.11, −0.01], *p*=.017).

When ERD, alexithymia, sex, age and ethnicity were covaried, inward expressions remained predictive of mental health problems (depression: *b*_IDE_=0.24, 95%CI [0.16,0.32]; anxiety: *b*_IDE_=0.30, 95%CI [0.23,0.37], both *p*s<0.0001), while outward distress expressions remained negatively associated with anxiety only (*b*_ODE_=−0.11, 95%CI [−0.16,−0.05], *p*<.0001). In these models, higher emotional burden, ERD, alexithymia, and age were also predictive of higher depression and anxiety (Full results in Supplementary Table S11).

### Group differences in the association between response after being upset and mental health

Associations between different responses after being upset and mental health problems mostly did not differ across groups, except for between solution-focused responses and anxiety. The ADHD groups (with or without autism) showed a stronger negative association between SFR score and anxiety compared to the control group (ADHD: *b* =−0.26, 95%CI [−0.46,−0.06], *p*=.012; combined: *b*=−0.25, 95%CI [−0.47,−0.04], *p*=.021), while the autism group did not (Fig. 2B). This pattern of relationship remained after covarying for alexithymia, sex, age, and ethnicity (see full results in Supplementary Materials S2, Table S12).

## Discussion

This study extends our previous exploration of the role of school-based emotional burden in predicting the likelihood of depression in autism and/or ADHD. We previously found that autistic and/or ADHD students experienced twice the school-based emotional burden as their control peers, which significantly correlated with depression and anxiety [11]. Here, we report how students’ responses after being upset might play a role in increasing this risk, using a novel, co-produced scale. The 20-item MERSI measures three intercorrelated constructs: *outward* and *inward distress expressions* and *solution-focused* *responses*. It has good overall internal consistency, excellent agreement between latent and domain scores, and satisfactory two-week test-retest reliability. Our analysis of the domain scores resulted in several findings of interest.

First, relative to the control group, the three neurodivergent groups presented different emotional responding profiles, despite similar levels of emotional burden. ADHD participants, irrespective of additional autism traits, presented with a different response profile from the autism-only group. Specifically, both ADHD groups were more likely to express distress both outwardly and inwardly, and less likely to deploy solution-focused responses, seemingly in line with the past findings of lower coping resources in ADHD [29, 30]. The distress expression in ADHD deviated from its common portrayal as primarily externalising [17, 21], presumably overshadowing their inward responses. Our findings highlight that, at least in the school context, ADHD adolescents have a strong inward component in their responses after being upset – including elevated levels of emotional masking or ruminative responses (i.e., “*hide your feelings*” and “*not be able to get it out of your mind*”). The coexistence of such seemingly incompatible responses should be further explored. Could they alternate within an individual rather than present at the same time, and be perceived as ‘emotional lability’ [64, 65] from the neurotypical lens? Could these responses be selectively associated with specific upsetting events? For instance, ADHD students might express distress inwardly to avoid punitive consequences or to ‘fit in’ with peers [7, 66, 67]. Lastly, could these expressions suggest sensitivity and priority to immediately reduce environmental provocations in ADHD rather than lower coping resources per se? Such possibilities are not examinable from the current data and should be considered in future studies.

The autism-only group, who, compared to the control group, had higher outward and inward distress expressions, demonstrated more prominence in inward distress expression relative to both ADHD groups. This was evidenced by lower outward expressions and highly ranked inward distress expression items, relative to the other neurodivergent groups. Inward expression of distress was higher in females than in males, and higher in White participants than those from minoritised ethnic groups. However, neurodivergent students still exhibited greater inward distress expression than controls after adjusting for differences in age, sex, and ethnicity across groups. Such a disparity between inward and outward distress expressions is present in the autism-only group despite their comparable level of solution-focused responses with the control peers. The tendency for inward distress expressions, compounded by efforts to execute solution-focused responses at school, arguably could lead to overwhelming internal processes, which, in combination with reduced outward expressions of distress, might underpin what parents describe as ‘autistic masking’ at school, which is, inevitably, ‘released’ at home [68–70].

Second, responses after being upset contributed to and improved the prediction of both depression and anxiety by emotional burden in young people. Among the different responses, while controlling for age, sex, ethnicity, and alexithymia, inward distress expression emerged as the strongest predictor for depression and anxiety, while outward distress expressions and solution-focused responses predicted lower anxiety only, albeit at a lower strength to be considered significant clinically. Importantly, these responses contributed to the prediction of mental health problems independently of emotion regulation deficits, explaining additional variance beyond these difficulties. Follow-up analyses showed that the link between inward distress expressions and mental health problems persisted in all groups, highlighting its transdiagnostic significance.

Third, developing solution-focused responses as a secondary intervention strategy could be helpful, particularly for those with ADHD, given their least reliance on solution-focused responses across groups, and a distinct association between this response type and lower mental health problems for this group. No such association was found in the autism-only group, suggesting that lessening inward distress expressions and environmental provocation should remain the primary focus of preventative interventions targeting mental health difficulties in this group. We should not hastily conclude that solution-focused responses serve no purpose in autistic-only students. This group demonstrates engagement in these responses comparable to their control peers, indicating potential utility that remains uncaptured by this study.

Altogether, our findings suggest that reducing environmental provocations—and, consequently, the emotional burden on adolescents—as well as ameliorating inward distress expressions, could be key targets for interventions aimed at preventing/lowering risk for depression or anxiety in the secondary school student population. One specific challenge for those supporting young people in schools will be recognising inward distress expressions. The extent of such support needs could be assessed through a confidential, school-wide administration of the scale. Facilitating students’ awareness of these tendencies and encouraging them to communicate with a trusted person may also represent a helpful first step. Furthermore, when designing support provision, it is important to acknowledge that response patterns may constitute contextually adaptive strategies within environments organised around neurotypical norms (e.g., masking can serve as an effort to maintain safety or acceptance, while outward expressions may indicate overwhelm rather than poor regulation). Consequently, reducing distress expressions should not be the sole target of intervention.

Our co-production and neurodiversity-informed approach resulted in a measure with good reliability and validity in a large sample of secondary school students in England. The study sample was enriched for participants with elevated ADHD and/or autism traits and those with formal clinical diagnoses, which increased representation across the neurodivergent spectrum and enabled informative subgroup analyses. However, this approach may also have introduced additional heterogeneity—not fully accounted for in the analyses— including the presence of other undiagnosed conditions. Since the study focused on secondary school-attending students, the findings may not generalise to individuals who are out of school or mainstream education. The study sample could also be affected by self-selection bias. It is also worth noting that the effect of ethnicity could not be analysed fully due to the under-representation of some ethnic subgroups among the neurodivergent participants. A specific limitation of the MERSI is that the individual responses (items) do not map to specific incidents in school (e.g., bullying versus a conflict with a teacher). The scale also does not exhaustively capture neurodivergent-specific regulation strategies such as stimming, sensory activity or withdrawal, or special-interest engagement, which can serve as adaptive self-regulation functions. Additionally, the MERSI does not specify a timeframe, which would be required to track changes over time. Most importantly, no conclusions can be drawn regarding the direction of effects in this cross-sectional study. It is plausible, for instance, that mental health problems drive increased levels of distress expressions instead. We are currently testing this longitudinally [71].

In summary, our findings show that neurodivergent and control individuals respond differently after being upset, and that the autism-only group responds differently from the ADHD group, irrespective of additional autism traits. In general, higher inward distress expressions predict higher depression and anxiety, alongside emotional burden, in all students, and thus ameliorating inward distress expressions could be one key focus for intervention design aimed at preventing/lowering the likelihood of depression or anxiety among secondary school students. Specifically for ADHD students, higher levels of solution-focused responses may also be helpful to lower mental health problems. These relationships are being explored further in a longitudinal study within the RE-STAR research programme.

## Supplementary Information

Below is the link to the electronic supplementary material.

Supplementary Material 1

## Data Availability

The data that support the findings of this study are available from the corresponding author upon reasonable request.

## References

[1] Shore L, Toumbourou JW, Lewis AJ, Kremer P (2018) Review: Longitudinal trajectories of child and adolescent depressive symptoms and their predictors - a systematic review and meta-analysis. Child Adolesc Ment Health 23:107–120. 10.1111/camh.12220 32677332

[2] Lai MC, Kassee C, Besney R, Bonato S, Hull L, Mandy W, Szatmari P, Ameis SH (2019) Prevalence of co-occurring mental health diagnoses in the autism population: a systematic review and meta-analysis. Lancet Psychiatry 6:819–829. 10.1016/S2215-0366(19)30289-5 31447415

[3] Danielson ML, Bitsko RH, Ghandour RM, Holbrook JR, Kogan MD, Blumberg SJ (2018) Prevalence of Parent-Reported ADHD Diagnosis and Associated Treatment Among U.S. Children and Adolescents, 2016. J Clin Child Adolesc Psychol 47:199–212. 10.1080/15374416.2017.1417860 PMC5834391 29363986

[4] Dow D, Morgan L, Hooker JL, Michaels MS, Joiner TE, Woods J, Wetherby AM (2021) Anxiety, depression, and the Interpersonal Theory of Suicide in a community sample of adults with Autism Spectrum Disorder. Arch Suicide Res 25:297–314. 10.1080/13811118.2019.1678537 31656121

[5] Wilens T, Nierenberg AA, Rostain A, Spencer TJ (2008) Adult attention-deficit hyperactivity disorder and the role of depression. CNS Spectr 13:2–17. 10.1017/S1092852900002935

[6] Factor RS, Ryan SM, Farley JP, Ollendick TH, Scarpa A (2017) Does the Presence of Anxiety and ADHD Symptoms Add to Social Impairment in Children with Autism Spectrum Disorder? J Autism Dev Disord 47:1122–1134. 10.1007/s10803-016-3025-9 28132125

[7] Pavlopoulou G, Chandler S, Lukito S, Kakoulidou M, Jackson I, Ly E, Matejko M, Balwani B, Boyens T, Poulton D, Harvey-Nguyen L, Baker S, Sonuga-Barke EJS (2025) Upsetting experiences in the lives of neurodivergent young people: a qualitative analysis of accounts of adolescents diagnosed with ADHD and/or autism. JCPP Adv e70038. 10.1002/jcv2.70038 PMC13260670 42291673

[8] Pavlopoulou G, Chandler S, Lukito S, Kakoulidou M, Matejko M, Jackson I, Balwani B, Boyens T, Poulton D, Harvey-Nguyen L, Glen Z, Wilson A, Ly E, Macauley E, Hurry J, Baker S, Sonuga-Barke EJS (2025) Situating emotion regulation in autism and ADHD through neurodivergent adolescents’ perspectives. Sci Rep 15:37464. 10.1038/s41598-025-21208-x PMC12559169 41145627

[9] Kakoulidou M, Pavlopoulou G, Chandler S, Lukito S, Matejko M, Jackson I, Balwani B, Boyens T, Poulton D, Harvey-Nguyen L, Glen Z, Wilson A, Ly E, Macauley E, Baker S, Bullen G, Sonuga-Barke EJS, team R-S (2024) Deepening the participation of neurodivergent youth in qualitative mental health research: co-development of a general approach and the evaluation of its implementation in a study on emotion. JCPP Adv 4:e12287. 10.1002/jcv2.12287 PMC11669790 39734931

[10] Sonuga-Barke EJS, Chandler S, Lukito S, Kakoulidou M, Moore G, Cooper N, Matejko M, Jackson I, Balwani B, Boyens T, Poulton D, Harvey-Nguyen L, Baker S, Pavlopoulou G (2024) Participatory translational science of neurodivergence: model for attention-deficit/hyperactivity disorder and autism research. Br J Psychiatry 224:127–131. 10.1192/bjp.2023.151 PMC10933558 38362636

[11] Lukito S, Chandler S, Kakoulidou M, Griffiths K, Wyatt A, Funnell E, Pavlopoulou G, Baker S, Stahl D, Sonuga-Barke E, RE-STAR team (2025) Emotional burden in school as a source of mental health problems associated with ADHD and/or autism: Development and validation of a new co-produced self-report measure. J Child Psychol Psychiatry 66(10):1577–1592. 10.1111/jcpp.70003 PMC12447693 40707015

[12] Victor SE, Klonsky ED (2016) Validation of a brief version of the Difficulties in Emotion Regulation Scale (DERS-18) in five samples. J Psychopathol Behav Assess 38:582–589. 10.1007/s10862-016-9547-9 PMC4882111 27239096

[13] Schäfer J, Naumann E, Holmes EA, Tuschen-Caffier B, Samson AC (2017) Emotion Regulation Strategies in Depressive and Anxiety Symptoms in Youth: A Meta-Analytic Review. J Youth Adolesc 46:261–276. 10.1007/s10964-016-0585-0 27734198

[14] Young KS, Sandman CF, Craske MG (2019) Positive and negative emotion regulation in adolescence: links to anxiety and depression. Brain Sci 9:76. 10.3390/brainsci9040076 PMC6523365 30934877

[15] Gross JJ (2015) Emotion Regulation: Current Status and Future Prospects. Psychol Inq 26:1–26. 10.1080/1047840X.2014.940781

[16] Christiansen H, Hirsch O, Albrecht B, Chavanon M-L (2019) Attention-Deficit/Hyperactivity Disorder (ADHD) and Emotion Regulation Over the Life Span. Curr Psychiatry Rep 21:17. 10.1007/s11920-019-1003-6 30826879

[17] Faraone SV, Rostain AL, Blader J, Busch B, Childress AC, Connor DF, Newcorn JH (2019) Practitioner review: Emotional dysregulation in attention-deficit/hyperactivity disorder – implications for clinical recognition and intervention. J Child Psychol Psychiatry 60:133–150. 10.1111/jcpp.12899 29624671

[18] Cai RY, Richdale AL, Uljarević M, Dissanayake C, Samson AC (2018) Emotion regulation in autism spectrum disorder: where we are and where we need to go. Autism Res 11:962–978. 10.1002/aur.1968 29979494

[19] Cibralic S, Kohlhoff J, Wallace N, McMahon C, Eapen V (2019) A systematic review of emotion regulation in children with autism spectrum disorder. Res Autism Spectr Disord 68:101422. 10.1016/j.rasd.2019.101422

[20] McDonald RG, Cargill MI, Khawar S, Kang E (2024) Emotion dysregulation in autism: A meta-analysis. Autism 28:2986–3001. 10.1177/13623613241257605 39080988

[21] Bunford N, Evans SW, Wymbs F (2015) ADHD and Emotion Dysregulation Among Children and Adolescents. Clin Child Fam Psychol Rev 18:185–217. 10.1007/s10567-015-0187-5 26243645

[22] Lipsky D (2011) From Anxiety to Meltdown: How Individuals on the Autism Spectrum Deal with Anxiety, Experience Meltdowns, Manifest Tantrums, and How You Can Intervene Effectively. Jessica Kingsley Publishers, London

[23] Belek B (2019) Articulating Sensory Sensitivity: From Bodies with Autism to Autistic Bodies. Med Anthropol 38:30–43. 10.1080/01459740.2018.1460750 29727204

[24] Lewis LF, Stevens K (2023) The lived experience of meltdowns for autistic adults. Autism 27:1817–1825. 10.1177/13623613221145783 36632658

[25] Patel S, Day TN, Jones N, Mazefsky CA (2016) Association between anger rumination and autism symptom severity, depression symptoms, aggression, and general dysregulation in adolescents with autism spectrum disorder. Autism 21:181–189. 10.1177/1362361316633566 PMC6070295 27095831

[26] Zimmer-Gembeck MJ, Skinner EA (2016) The development of coping: Implications for psychopathology and resilience. Developmental psychopathology: Risk, resilience, and intervention, vol Vol. 4, 3rd ed. John Wiley & Sons, Inc., Hoboken, NJ, US, p p 485–545

[27] Mazefsky CA, Borue X, Day TN, Minshew NJ (2014) Emotion regulation patterns in adolescents with high-functioning autism spectrum disorder: comparison to typically developing adolescents and association with psychiatric symptoms. Autism Res 7:344–354. 10.1002/aur.1366 PMC4136477 24610869

[28] Samson AC, Hardan AY, Lee IA, Phillips JM, Gross JJ (2015) Maladaptive Behavior in Autism Spectrum Disorder: The Role of Emotion Experience and Emotion Regulation. J Autism Dev Disord 45:3424–3432. 10.1007/s10803-015-2388-7 25711546

[29] Mayer JS, Brandt GA, Medda J, Basten U, Grimm O, Reif A, Freitag CM (2022) Depressive symptoms in youth with ADHD: the role of impairments in cognitive emotion regulation. Eur Arch Psychiatry Clin NeuroSci 272:793–806. 10.1007/s00406-022-01382-z PMC9279209 35107603

[30] Ringer N (2019) Young people’s perceptions of and coping with their ADHD symptoms: A qualitative study. Cogent Psychol 6. 10.1080/23311908.2019.1608032

[31] Strohmeier CW, Rosenfield B, DiTomasso RA, Ramsay JR (2016) Assessment of the relationship between self-reported cognitive distortions and adult ADHD, anxiety, depression, and hopelessness. Psychiatry Res 238:153–158. 10.1016/j.psychres.2016.02.034 27086226

[32] Clarbour J, Roger D (2004) The construction and validation of a new scale for measuring emotional response style in adolescents. J Child Psychol Psychiatry 45:496–509. 10.1111/j.1469-7610.2004.00240.x 15055369

[33] Achenbach TM, Rescorla L (2001) Manual for the ASEBA school-age forms & profiles: an integrated system of multi-informant assessment. ASEBA, Burlington, VT

[34] Stringaris A, Goodman R, Ferdinando S, Razdan V, Muhrer E, Leibenluft E, Brotman MA (2012) The Affective Reactivity Index: a concise irritability scale for clinical and research settings. J Child Psychol Psyc 53:1109–1117. 10.1111/j.1469-7610.2012.02561.x PMC3484687 22574736

[35] Nock MK, Wedig MM, Holmberg EB, Hooley JM (2008) The emotion reactivity scale: development, evaluation, and relation to self-injurious thoughts and behaviors. Behav Ther 39:107–116. 10.1016/j.beth.2007.05.005 18502244

[36] Mazefsky CA, Yu L, White SW, Siegel M, Pilkonis PA (2018) The emotion dysregulation inventory: Psychometric properties and item response theory calibration in an autism spectrum disorder sample. Autism Res 11:928–941. 10.1002/aur.1947 PMC6026073 29624893

[37] Mehta A, Moeck E, Preece DA, Koval P, Gross JJ (2025) Alexithymia and Emotion Regulation: the Role of Emotion Intensity. Affect Sci 6:77–93. 10.1007/s42761-024-00278-6 PMC11904038 40094046

[38] Preece DA, Mehta A, Petrova K, Sikka P, Bjureberg J, Becerra R, Gross JJ (2023) Alexithymia and emotion regulation. J Affect Disord 324:232–238. 10.1016/j.jad.2022.12.065 36566943

[39] Maiano C, Normand CL, Salvas MC, Moullec G, Aime A (2016) Prevalence of school bullying among youth with autism spectrum disorders: A systematic review and meta-analysis. Autism Res 9:601–615. 10.1002/aur.1568 26451871

[40] Simmons JA, Antshel KM (2021) Bullying and depression in youth with ADHD: A systematic review. Child Youth Care Forum. 50:379–414. 10.1007/s10566-020-09586-x

[41] MacLean J, Krause A, Rogers MA (2023) The student-teacher relationship and ADHD symptomatology: a meta-analysis. J Sch Psychol 99:101217. 10.1016/j.jsp.2023.04.007 37507182

[42] Costley D, Emerson A, Ropar D, Sheppard E (2021) The anxiety caused by secondary schools for autistic adolescents: In their own words. Educ Sci 11:726. 10.3390/educsci11110726

[43] Rutter M (2003) The Social Communication Questionnaire: Manual. Western Psychological Services

[44] Swanson JM, Kraemer HC, Hinshaw SP, Arnold LE, Conners CK, Abikoff HB, Clevenger W, Davies M, Elliott GR, Greenhill LL (2001) Clinical relevance of the primary findings of the MTA: success rates based on severity of ADHD and ODD symptoms at the end of treatment. J Am Acad Child Adolesc Psychiatry 40:168–179. 10.1097/00004583-200102000-00011 11211365

[45] Bussing R, Fernandez M, Harwood M, Wei H, Garvan CW, Eyberg SM, Swanson JM (2008) Parent and teacher SNAP-IV ratings of attention deficit hyperactivity disorder symptoms: psychometric properties and normative ratings from a school district sample. Assessment 15:317–328. 10.1177/1073191107313888 PMC3623293 18310593

[46] Fonseca-Pedrero E, Díez-Gómez A, Pérez-Albéniz A, Al-Halabí S, Lucas-Molina B, Debbané M (2023) Youth screening depression: Validation of the Patient Health Questionnaire-9 (PHQ-9) in a representative sample of adolescents. Psychiatry Res 328:115486. 10.1016/j.psychres.2023.115486 37738682

[47] Mossman SA, Luft MJ, Schroeder HK, Varney ST, Fleck DE, Barzman DH, Gilman R, DelBello MP, Strawn JR (2017) The Generalized Anxiety Disorder 7-item (GAD-7) scale in adolescents with generalized anxiety disorder: signal detection and validation. Annals Clin Psychiatry: official J Am Acad Clin Psychiatrists 29:227–234. 10.1177/104012371702900404 PMC5765270 29069107

[48] Bagby RM, Parker JDA, Taylor GJ (1994) The twenty-item Toronto Alexithymia scale—I. Item selection and cross-validation of the factor structure. J Psychosom Res 38:23–32. 10.1016/0022-3999(94)90005-1 8126686

[49] Arifin WN (2018) A Web-based Sample Size Calculator for Reliability Studies. Educ Med J 10:67–76. 10.21315/eimj2018.10.3.8

[50] Revelle W, Evanston (2024) psych: Procedures for Psychological, Psychometric, and Personality Research, R package version 2.4.1. 10.32614/CRAN.package.psych

[51] Rosseel Y (2012) lavaan: An R Package for Structural Equation Modeling. J Stat Softw 48:1–36. 10.18637/jss.v048.i02

[52] Ripley B, Venables W, Bates DM, Hornik K, Gebhardt A, Firth D MASS: Support Functions and Datasets for Venables and Ripley’s Modern Applied Statistics with S 4th Edition. University of Oxford, Kingdom U (2024) , R package version 7.3–61. http://www.stats.ox.ac.uk/pub/MASS4/

[53] Gamer M, Lemon J, Fellows I, Singh P, Germany (2022) Package‘irr’ : Various Coefficients of Interrater Reliabilityand Agreement. R package version 0.8. https://cran.r-project.org/web/packages/irr/irr.pdf

[54] Canty A, Ripley B (2025) Bootstrap Functions, R package version 1:3–32. https://cran.r-project.org/web/packages/boot/boot.pdf

[55] Schafer JL (1999) Multiple imputation: a primer. Stat Methods Med Res 8:3–15. 10.1177/096228029900800102 10347857

[56] Cattell RB (1966) The Scree Test For The Number Of Factors. Multivar Behav Res 1:245–276. 10.1207/s15327906mbr0102_10 26828106

[57] Horn JL (1965) A rationale and test for the number of factors in factor analysis. Psychometrika 30:179–185. 10.1007/BF02289447 14306381

[58] Costello AB, Osborne JW (2005) Best practices in exploratory factor analysis: four recommendations for getting the most from your analysis. Practical Assess Res Evaluation 10:1–9. 10.7275/jyj1-4868

[59] Streiner DL, Norman GR (2008) Health Measurement Scales : A practical guide to their development and use. Oxford University Press, Incorporated

[60] Bentler PM, Bonett DG (1980) Significance tests and goodness of fit in the analysis of covariance structures. Psychol Bull 88:588. 10.1037/0033-2909.88.3.588

[61] Schwarz G (1978) Estimating the Dimension of a Model. Annals Stat 6:461–464. 10.1214/aos/1176344136

[62] Davison A, Hinkley D (1997) Bootstrap Methods and Their Application. J Am Stat Assoc 94:533. 10.1017/CBO9780511802843

[63] Chen FF (2007) Sensitivity of Goodness of Fit Indexes to Lack of Measurement Invariance. Struct Equation Modeling: Multidisciplinary J 14:464–504. 10.1080/10705510701301834

[64] Maire J, Galera C, Bioulac S, Bouvard M, Michel G (2020) Emotional lability and irritability have specific associations with symptomatology in children with attention deficit hyperactivity disorder. Psychiatry Res 285:112789. 10.1016/j.psychres.2020.112789 32014627

[65] Van Liefferinge D, Sonuga-Barke EJS, Danckaerts M, Van Broeck N, van der Oord S (2021) Do Childhood Emotional Lability and ADHD Symptoms Have Shared Neuropsychological Roots? J Psychopathol Behav Assess 43:491–505. 10.1007/s10862-020-09859-8

[66] Gibbs K, Carrington S, Mercer KL (2022) Perspectives about Friendships and the School Learning Environment from Australian Adolescent Boys with AD/HD. Int J Disabil Dev Educ 69:1974–1987. 10.1080/1034912X.2020.1840529

[67] Gibbs K, Mercer KL, Carrington S (2016) The Schooling Experience of Adolescent Boys with AD/HD: An Australian Case Study. Int J Disabil Dev Educ 63:608–622. 10.1080/1034912X.2016.1164302

[68] Hill C, Keville S, Ludlow AK (2023) Inclusivity for children with autism spectrum disorders: Parents’ reflections of the school learning environment versus home learning during COVID-19. Int J Dev Disabil 69:546–554. 10.1080/20473869.2021.1975253 PMC10281296 37346262

[69] Carrington S, Graham L (2001) Perceptions of school by two teenage boys with Asperger syndrome and their mothers: a qualitative study. Autism 5:37–48. 10.1177/1362361301005001004 11708388

[70] Sciutto M, Richwine S, Mentrikoski J, Niedzwiecki K (2012) A Qualitative Analysis of the School Experiences of Students With Asperger Syndrome. Focus Autism Other Dev Disabil 27:177–188. 10.1177/1088357612450511

[71] Sonuga-Barke EJS, Palmer M, Griffiths K, Wyatt A, Danese A, Chandler S, Stahl D, Lukito S, Pavlopoulou G, Simonoff E (2025) Why do attention-deficit/hyperactive disorder and/or autism traits place adolescents at risk for depression? Protocol for a longitudinal comparison of the mediating role of deficits in emotional processing and control versus emotional burden. JCPP Adv. 10.1002/jcv2.70052 PMC12973166 41815767

