# Supplementary material for: Autistic and ADHD adolescents respond differently to upset in school: evidence from a new questionnaire co-designed from the neurodivergent point of view
Source: Eur Child Adolesc Psychiatry. 2026 Jun 1;35(9):2977–90. doi: 10.1007/s00787-026-03073-9 (PMC13695674; doi:10.1007/s00787-026-03073-9)
Supplement: Supplementary file 1 [file 787_2026_3073_MOESM1_ESM.docx]

**Supplementary materials to Lukito*, Chandler* et al. (2026). Autistic and ADHD adolescents respond differently to upset in school: Evidence from a new questionnaire co-designed from the neurodivergent point of view.**

* Joint first authors

**Table of Content**

[S1. SUPPLEMENTARY METHODS	2](#_Toc224825162)

[The My Emotional Responses in School Inventory (MERSI) Prototype	2](#_Toc224825163)

[Preliminary Item Analysis Criteria	2](#_Toc224825164)

[Exploratory Factor Analysis	3](#_Toc224825165)

[Measurement Equivalence Analysis	3](#_Toc224825166)

[Correlation between MERSI and DERS Domain Scores	3](#_Toc224825167)

[MERSI Domain Scores and Formal Diagnosis Status	3](#_Toc224825168)

[Adjusted Group Differences of MERSI Responses	3](#_Toc224825169)

[Adjusted Cross-sectional Prediction of Depression and Anxiety	3](#_Toc224825170)

[Modification of Adjusted Association between MERSI Domain Scores and Mental Health Problems by Group	4](#_Toc224825171)

[S2. SUPPLEMENTARY RESULTS	5](#_Toc224825172)

[Preliminary Item Analysis	5](#_Toc224825173)

[Exploratory Factor Analysis (EFA) Findings	6](#_Toc224825174)

[Scree and Parallel Analysis Plot	6](#_Toc224825175)

[Initial EFA	6](#_Toc224825176)

[Final EFA	7](#_Toc224825177)

[Measurement Equivalence	11](#_Toc224825178)

[Correlation between MERSI and DERS Domain Scores	12](#_Toc224825179)

[The Influences of Formal Diagnosis Status on MERSI Scores	13](#_Toc224825180)

[Adjusted Group Differences of MERSI Responses	14](#_Toc224825181)

[Adjusted Cross-sectional Prediction of Depression or Anxiety Using the MERSI Domain Scores	15](#_Toc224825182)

[Group Differences in the Adjusted Cross-sectional Prediction of Mental Health Problems	15](#_Toc224825183)

[REFERENCES	16](#_Toc224825184)

# S1. SUPPLEMENTARY METHODS

## The My Emotional Responses in School Inventory (MERSI) Prototype

The prototype of MERSI contains 24 items reflecting different expressions or responses that adolescents exhibit after experiencing an upset at school (Table S1). The likelihood of each response is rated on a five-point Likert scale (0=*very unlikely*, 1=*unlikely*, 2=*possibly*, 3=*likely*, 4=*very likely*). Four items were dropped during the psychometric scale development stage, leaving 20 items in the final MERSI.

**Table S1. The MERSI Prototype Items**

| We would like to know **what you would do** if something made you extremely upset at school. | | | | | |
| --- | --- | --- | --- | --- | --- |
| 1. | Shout and/or swear (either or both). | | | | |
|  | Very unlikely | Unlikely | Possibly | Likely | Very likely |
|  | ☐ | ☐ | ☐ | ☐ | ☐ |
| 2. | Hit out/punch objects. | | | | |
| 3. | Get into a fight. | | | | |
| 4. | Storm off. | | | | |
| 5. | Withdraw into yourself, shut everything out. | | | | |
| 6. | Hide your feelings. | | | | |
| 7. | Hurt yourself. | | | | |
| 8. | Not be able to get it out of your mind. | | | | |
| 9. | Confront the person/situation. | | | | |
| 10. | Get into an argument. | | | | |
| 11. | Leave the situation to find your own space. | | | | |
| 12. | Talk to an adult. | | | | |
| 13. | Do something you find comforting (put headphones in, use a fidget toy, or other sensory activity). | | | | |
| 14. | Re-think things, not blow things out of proportion. | | | | |
| 15. | Try not to think about it, and let it pass. | | | | |
| 16. | Distract yourself by thinking about positive things. | | | | |
| 17. | Look for support from others. | | | | |
| 18. | Look for explanations for why the situation has occurred. | | | | |
| 19. | Neglect your daily routine (e.g., stop sleeping or eating well). | | | | |
| 20. | Hold a grudge. | | | | |
| 21. | Use strategies you’ve ‘learnt’ to manage your feelings (e.g., taking deep breaths, counting down). | | | | |
| 22. | Feel physically sick (e.g., have a headache, stomach pain). | | | | |
| 23. | Refuse to do what a school staff member asks you to do. | | | | |
| 24. | Switch or swing from one emotion to another. | | | | |

Note. The prototype of MERSI contains 24 items, four of which (Item numbers 11,13,19,20) are excluded from the final scale

## Preliminary Item Analysis Criteria

We initially established whether the MERSI prototype response items: (a) had sufficient variation and were *not* rated the same way by >80% of participants, (b) demonstrated no signs of separate distributions, e.g., bimodal across the control and neurodivergence subgroups, (c) were not pairwise-intercorrelated above *r*>0.8 and had at least one correlation with *r*>0.3 with other items [see 1], (d) had less than 5% missing responses, and (e) had absolute two-week test-retest agreement >0.4 to consider their removal.

## Exploratory Factor Analysis

MERSI structure was investigated using exploratory factor analysis (EFA) with ordinary least squares, applying *Oblimin* rotation for correlated factors. Scree plots [2] and parallel analyses [3] helped identify optimal factor number. Items were considered for removal if they: loaded on a factor with fewer than five indicators, unless their loadings were >0.7 [4]; loaded with a factor loading <0.4; or cross-loaded in more than one factor with a secondary loading of ≥0.3, or with a loading difference <0.2 [1]. Model selection was aided by goodness-of-fit criteria, including likelihood ratio chi-square, Tucker-Lewis Indices (TLI; good fit ≥.95 [5]), and the Bayesian information criterion (BIC) [6]. The model's robustness was further examined using the bias-corrected bootstrap for skewed responses [7].

## Measurement Equivalence Analysis

As an additional robustness analysis, we examined the measurement equivalence or invariance of the MERSI domain factors using a series of multi-group confirmatory factor analyses (MGCFA). We examined the measurement model consistency across groups (i.e., ADHD, autism, combined, and comparison), biological sex (male, female), and ethnicity (white, minoritised ethnic groups). Three levels of invariance were tested: (1) the configural invariance was evaluated to determine whether the same factor structure was applicable across groups, (2) the metric invariance was tested by constraining factor loadings to be equal across groups, indicating that the constructs were measured in the same way between groups, and (3) the scalar invariance was tested by constraining item intercepts to equality across groups, allowing unbiased comparison of latent means between groups. However, scalar invariance was not expected for the MERSI, as attitudinal measures often exhibit group differences in item intercepts. Invariance was evaluated using changes in fit indices (ΔCFI ≤ .005, ΔRMSEA ≤ .010), following recommended thresholds for unequal group sizes below *n* = 300 [8]

## Correlation between MERSI and DERS Domain Scores

To examine the convergence between MERSI and DERS domain scores, Pearson’s pairwise correlation coefficient was computed and presented in a correlation matrix. Pearson’s *r* effect size values were interpreted as < 0.30 (small), 0.30–0.49 (medium), 0.50–0.79 (large), 0.80–1.0 (very large), adapted from a previous guideline [9]

## MERSI Domain Scores and Formal Diagnosis Status

To examine whether a formal diagnosis status might be associated with differences in MERSI scores among those assigned into the ADHD, autism, and combined ADHD+autism groups, an additional full-factorial 2(formal diagnosis status) x 3(group) ANOVA was conducted. Partial eta squared (*η*_p_²) was reported as the effect size for the ANOVA *F*-statistics (<0.010 [very small], 0.01–0.059 [small], 0.06–0.139 [medium], ≥0.14 [large]), while adjusted Cohen’s *d* was used as the effect size for the pairwise comparisons (<0.20 [very small], 0.20–0.49 [small], 0.50–0.79 [medium], ≥0.80 [large]) [10]. *Post hoc* pairwise comparisons of estimated marginal means were conducted using Tukey adjustment.

## Adjusted Group Differences of MERSI Responses

We examined if group differences of MERSI responses remained after adjusting for age, sex, and ethnicity (white vs. minoritised ethnic group), since these variables differed across groups. To test for violation of the homogeneity of regression slope assumption, we also added interaction terms between these variables and the participant group. Partial eta squared and adjusted Cohen’s *d* were reported following the guideline above. *Post hoc* pairwise comparisons of estimated marginal means were conducted using Tukey adjustment.

## Adjusted Cross-sectional Prediction of Depression and Anxiety

The associations between MERSI domain scores and mental health problems (depression or anxiety) were further examined while controlling for confounders (i.e., age, sex, ethnicity, and alexithymia) as well as including EB and ERD. The ERD was included in the model to assess the independent contributions of EB and responses after being upset from emotional dysregulation, as we have previously shown the unique contribution of EB and ERD in predicting depression and anxiety in our previous study [11]. Standardised beta (*b*) was used as an effect size measure in these regression analyses.

## Modification of Adjusted Association between MERSI Domain Scores and Mental Health Problems by Group

To explore whether the relationship between the domain scores and depression or anxiety is modified by group, we added interaction terms between Group x MERSI domain scores in the simple linear regression between each MERSI domain score and depression or anxiety. We adjusted the association by adding the confounders (i.e., age, sex, ethnicity, and alexithymia).

# S2. SUPPLEMENTARY RESULTS

## Preliminary Item Analysis

No items were given the same score by over 80% of participants (Range = 21.3-58.4%). The items that scored most similarly among the participants were items 7 (*‘Hurt yourself’*; 58.4%) and 3 (*‘Get into a fight’*; 54.4%), to which the answer ‘Very unlikely’ was the most common. No items were bimodally distributed (Fig. S1). No two items correlated with *r* > 0.8 (Range = -0.28-0.57. All items had at least one correlation *r*>0.3 with other items (Range = 13-22 other items). Percent missing responses per item ranged between 0-1%.

**Figure S1 Distribution of MERSI items**

Notable preliminary item analysis findings included the low item-level two-week test-retest agreement |ICC| < 0.1 for item 13 *“Do something you find comforting (put headphones in, use a fidget toy, or other sensory activity”*, and poor two-week test-retest agreement |ICC| < 0.4 for items 11 *“Leave the situation to find your own space”* and 16 *“Distract yourself by thinking about positive things”* (Table S2). Nevertheless, these items were entered into the Exploratory Factor Analysis.

**Table S2. Two-Week Test-retest Agreement**

| **Item #** | **df1** | | **df2** | **ICC 95% CI** | **p-value** |
| --- | --- | --- | --- | --- | --- |
| 1 | 45 | 45 | | 0.487 [0.232, 0.679] | <.001*** |
| 2 | 45 | 45 | | 0.481 [0.224, 0.675] | <.001*** |
| 3 | 45 | 45 | | 0.614 [0.396, 0.766] | <.001*** |
| 4 | 45 | 45 | | 0.423 [0.154, 0.634] | .002** |
| 5 | 45 | 45 | | 0.622 [0.407, 0.771] | <.001*** |
| 6 | 45 | 45 | | 0.520 [0.274, 0.703] | <.001*** |
| 7 | 45 | 45 | | 0.832 [0.716, 0.904] | <.001*** |
| 8 | 44 | 44 | | 0.470 [0.208, 0.670] | <.001*** |
| 9 | 45 | 45 | | 0.700 [0.516, 0.822] | <.001*** |
| 10 | 45 | 45 | | 0.407 [0.135, 0.622] | .002** |
| 11 | 45 | 45 | | 0.292 [0.005, 0.534] | .023* |
| 12 | 45 | 45 | | 0.442 [0.177, 0.648] | <.001*** |
| 13 | 45 | 45 | | -0.047 [-0.330, 0.244] | .623 |
| 14 | 45 | 45 | | 0.530 [0.287, 0.710] | <.001*** |
| 15 | 45 | 45 | | 0.484 [0.228, 0.677] | <.001*** |
| 16 | 45 | 45 | | 0.347 [0.066, 0.577] | .008** |
| 17 | 45 | 45 | | 0.732 [0.563, 0.842] | <.001*** |
| 18 | 45 | 45 | | 0.419 [0.150, 0.631] | .002** |
| 19 | 45 | 45 | | 0.662 [0.462, 0.797] | <.001*** |
| 20 | 45 | 45 | | 0.695 [0.509, 0.819] | <.001*** |
| 21 | 45 | 45 | | 0.528 [0.284, 0.708] | <.001*** |
| 22 | 45 | 45 | | 0.576 [0.345, 0.741] | <.001*** |
| 23 | 45 | 45 | | 0.651 [0.448, 0.791] | <.001*** |
| 24 | 45 | 45 | | 0.408 [0.137, 0.623] | .002** |
| Note. p-values *** <.001, ** <.01, * <.05 | | | | | |

## Exploratory Factor Analysis (EFA) Findings

### Scree and Parallel Analysis Plot

The scree and parallel analysis plots strongly suggested a three-factor structure for the MERSI items (Figure S2), with a potential emergence of a weaker fourth factor.

**Figure S2. Scree and Parallel Analysis Plots**

| (a) | (b) |
| --- | --- |
|  |  |
| Note. Eigenvalues or variance explained by each principal component (PC) from the principal component analysis and factor (FA) from the factor analysis are plotted in the scree plot (a). Only eigenvalues from the PC of actual data (blue) are plotted together in a parallel analysis plot (b), presented with plots of random eigenvalues or a simulated data line (red). | |

### Initial EFA

The initial EFA showed two items with factor loadings < .40: Item 13 *“Do something you find comforting (put headphones in, use a fidget toy or other sensory activity)”* and item 20 “*Hold a grudge”*. Item 13 had been found to have a low two-week test-retest reliability (|ICC|<.1) (Table S3). A third item 11 *“Leave the situation to find your own space”*, cross-loaded on two latent factors (factor loadings 0.39 and 0.44, Table S3). One further item, “*Neglect your daily routine (e.g., stop sleeping or eating well)”,* was deemed to have a longer time frame than other responses (loading=0.67). These items were removed from the final MERSI scale.

**Table S3. Loading of Indicators in the Initial EFA with a Three-Factor Solution**

| **Item #** | **MR1** | **MR2** | **MR3** | **h2** | **u2** | **Com** |
| --- | --- | --- | --- | --- | --- | --- |
| 1 | 0.72 | 0.01 | -0.07 | 0.546 | 0.454 | 1.021 |
| 2 | 0.65 | 0.11 | -0.03 | 0.489 | 0.511 | 1.064 |
| 3 | 0.70 | -0.06 | -0.07 | 0.493 | 0.507 | 1.036 |
| 4 | 0.53 | 0.33 | 0.03 | 0.514 | 0.486 | 1.685 |
| 5 | 0.02 | 0.71 | 0.05 | 0.503 | 0.497 | 1.011 |
| 6 | -0.15 | 0.56 | -0.01 | 0.275 | 0.725 | 1.143 |
| 7 | 0.04 | 0.53 | -0.09 | 0.317 | 0.683 | 1.066 |
| 8 | 0.07 | 0.68 | -0.03 | 0.510 | 0.490 | 1.024 |
| 9 | 0.64 | -0.16 | 0.22 | 0.365 | 0.635 | 1.366 |
| 10 | 0.77 | -0.03 | 0.02 | 0.575 | 0.425 | 1.003 |
| 11* | -0.03 | 0.39 | 0.44 | 0.318 | 0.682 | 1.986 |
| 12 | 0.00 | -0.04 | 0.49 | 0.245 | 0.755 | 1.014 |
| 13* | -0.06 | 0.39 | 0.36 | 0.258 | 0.742 | 2.034 |
| 14 | -0.02 | -0.05 | 0.60 | 0.379 | 0.621 | 1.018 |
| 15 | -0.02 | -0.21 | 0.50 | 0.312 | 0.688 | 1.349 |
| 16 | -0.03 | -0.10 | 0.68 | 0.490 | 0.510 | 1.045 |
| 17 | 0.09 | -0.08 | 0.60 | 0.361 | 0.639 | 1.078 |
| 18 | 0.02 | 0.08 | 0.56 | 0.316 | 0.684 | 1.046 |
| 19* | 0.05 | 0.67 | -0.07 | 0.483 | 0.517 | 1.035 |
| 20* | 0.30 | 0.35 | -0.10 | 0.306 | 0.694 | 2.137 |
| 21 | -0.09 | 0.22 | 0.55 | 0.348 | 0.653 | 1.352 |
| 22 | -0.06 | 0.70 | 0.01 | 0.459 | 0.541 | 1.016 |
| 23 | 0.57 | 0.17 | -0.07 | 0.439 | 0.561 | 1.219 |
| 24 | 0.28 | 0.58 | -0.06 | 0.535 | 0.465 | 1.467 |
| Factor loadings of the MERSI prototype items are listed in the table, alongside their communality (h2) uniqueness (u2) and item complexity (com). Note. Items with asterisks were dropped from the final MERSI | | | | | | |

### Final EFA

The final 20-item MERSI contained three factors, with item-factor loadings *B*s=0.48-0.76, item communalities *h*2=0.23-0.56 (Table S4), and an acceptable fit (Likelihood Chi2 = 782, *p* <.001; TLI = 0.814; RMSEA = 0.082, 90% CI [0.076, 0.087]; BIC = -95.7; RMSR = 0.05) explaining 42% of variance in the data. The factors were labelled: (1) ‘*Outward Distress Expressions (ODE)’,* (2) ‘*Inward Distress Expressions’ (IDE)*, and (3) *Solution-Focused Responses (SFR)*. Factors 1 and 2 correlated moderately and positively (*r* = 0.35). Factors 1 and 3 (*r* = -0.19) and Factors 2 and 3 (*r* = -0.13) correlated weakly and negatively. The mean, 95% confidence interval, and rank of each MERSI item in the overall and each participant group are presented in Table S5.

**Table S4. Factor Loadings and Internal Consistency of the final MERSI Items**

|  | **B1 [95%CI]** | **B2 [95%CI]** | **B3 [95%CI]** | **h2** | **Raw α** | **Avg r** | **Raw r** | **Rdrop** |  |
| --- | --- | --- | --- | --- | --- | --- | --- | --- | --- |
| **Factor 1: Outward Distress Expressions (ODE)** | | | | | | | | | |
| Shout and/or swear (either or both). | **0.72 [0.67, 0.77]** | 0.00 [-0.08, 0.07] | -0.08 [-0.13, -0.01] | 0.545 | 0.820 | 0.436 | 0.773 | 0.664 |  |
| Hit out/punch objects. | **0.66 [0.60, 0.72]** | 0.11 [-0.01, 0.19] | -0.03 [-0.09, 0.05] | 0.501 | 0.822 | 0.439 | 0.755 | 0.652 |  |
| Get into a fight. | **0.71 [0.66, 0.77]** | -0.06 [-0.11, 0.01] | -0.06 [-0.12, -0.00] | 0.497 | 0.827 | 0.443 | 0.728 | 0.637 |  |
| Storm off. | **0.54 [0.46, 0.61]** | 0.31 [0.04, 0.40] | -0.01 [-0.08, 0.07] | 0.504 | 0.829 | 0.453 | 0.727 | 0.603 |  |
| Confront the person/situation | **0.63 [0.56, 0.69]** | -0.15 [-0.21, 0.19] | 0.21 [0.13, 0.28] | 0.356 | 0.850 | 0.490 | 0.610 | 0.461 |  |
| Get into an argument. | **0.76 [0.71, 0.80]** | -0.02 [-0.08, 0.03] | 0.02 [-0.04, 0.07] | 0.559 | 0.818 | 0.434 | 0.778 | 0.675 |  |
| Refuse to do what school staff member asks you to do. | **0.57 [0.49, 0.66]** | 0.17 [0.08, 0.27] | -0.06 [-0.13, 0.04] | 0.442 | 0.831 | 0.455 | 0.716 | 0.591 |  |
| **Factor 2: Inward Distress Expressions (IDE)** | | | | | | | | | |
| Withdraw into yourself, shut everything out. | 0.03 [-0.02, 0.65] | **0.70 [0.07, 0.76]** | 0.02 [-0.05, 0.08] | 0.490 | 0.762 | 0.392 | 0.755 | 0.612 |  |
| Hide your feelings. | -0.01 [-0.08, 0.10] | **0.56 [0.03, 0.64]** | -0.16 [-0.25, -0.07] | 0.278 | 0.796 | 0.438 | 0.641 | 0.466 |  |
| Hurt yourself. | -0.07 [-0.14, 0.02] | **0.53 [0.01, 0.61]** | 0.05 [-0.03, 0.15] | 0.321 | 0.788 | 0.427 | 0.642 | 0.498 |  |
| Not be able to get it out of your mind. | -0.02 [-0.07, 0.64] | **0.69 [0.61, 0.74]** | 0.06 [-0.00, 0.12] | 0.513 | 0.756 | 0.383 | 0.768 | 0.640 |  |
| Feel physically sick. | 0.03 [-0.02, 0.65] | **0.70 [0.06, 0.76]** | -0.06 [-0.12, 0.01] | 0.459 | 0.771 | 0.401 | 0.731 | 0.577 |  |
| Switch or swing from one emotion to another. | -0.05 [-0.11, 0.02] | **0.58 [0.06, 0.76]** | 0.28 [0.20, 0.36] | 0.533 | 0.769 | 0.400 | 0.729 | 0.585 |  |
| **Factor 3: Solution-Focused Responses (SFR)** | | | | | | | | | |
| Talk to an adult. | -0.02 [-0.11, 0.10] | 0.00 [-0.09, 0.10] | **0.48 [0.34, 0.55]** | 0.231 | 0.764 | 0.350 | 0.596 | 0.421 |  |
| Re-think things, not blow things out of proportion. | -0.02 [-0.09, 0.59] | -0.03 [-0.11, 0.04] | **0.60 [0.05, 0.67]** | 0.372 | 0.743 | 0.326 | 0.669 | 0.521 |  |
| Try not to think about it, and let it pass. | -0.19 [-0.28, 0.48] | -0.02 [-0.11, 0.06] | **0.50 [0.38, 0.58]** | 0.322 | 0.759 | 0.344 | 0.609 | 0.443 |  |
| Distract yourself by thinking about positive things. | -0.07 [-0.15, 0.19] | -0.03 [-0.09, 0.04] | **0.68 [0.59, 0.73]** | 0.490 | 0.726 | 0.306 | 0.735 | 0.606 |  |
| Look for support from others. | -0.03 [-0.11, 0.07] | 0.09 [0.01, 0.16] | **0.63 [0.54, 0.69]** | 0.392 | 0.739 | 0.322 | 0.687 | 0.541 |  |
| Look for explanations for why the situation has occurred. | 0.13 [0.05, 0.23] | 0.01 [-0.06, -0.00] | **0.60 [0.54, 0.69]** | 0.351 | 0.750 | 0.334 | 0.644 | 0.486 |  |
| Use strategies you’ve ‘learnt’ to manage your feelings (e.g., taking deep breaths, counting down). | 0.25 [0.17, 0.54] | -0.09 [-0.17, -0.00] | **0.55 [0.51, 0.67]** | 0.344 | 0.753 | 0.338 | 0.633 | 0.472 |  |

Note. Factor loadings, their bias-corrected bootstrapped 95% confidence interval (CI), and internal consistency of the final MERSI items within each subdomain. Abbreviation. MR = factor loadings based on minimum residuals, h2= communality, i.e., the proportion of variance of the common factors; Raw *α* = the value of internal consistency measure Cronbach’s *α* when a subdomain item was omitted; Avg *r* = average inter-item correlation between each item with all other items within the subdomain; Raw *r* = the item-total correlation, i.e., the correlation between each item, with the total scores including all items within the subdomain; *R*drop = the item-total correlation between each item with the total subdomain score, omitting that item from the subdomain score calculation.

**Table S5. Mean, Confidence Interval, and Mean Rank of the MERSI Items**

|  | Overall  (N=732) | | | ADHD  (n=100) | | | Autism  (n=100) | | | Combined  (n=79) | | | Control  (n=453) | | |
| --- | --- | --- | --- | --- | --- | --- | --- | --- | --- | --- | --- | --- | --- | --- | --- |
| (i) **MERSI Item Scores** | | | | | | | | | | | | | | | |
|  | M | 95% CI | Rank | M | 95% CI | Rank | M | 95% CI | Rank | M | 95% CI | Rank | M | 95% CI | Rank |
| [6] Hide your feelings. | 2.48 | [2.38, 2.58] | 1 | 2.59 | [2.32, 2.86] | 4 | 2.71 | [2.45, 2.97] | 2 | 2.62 | [2.30, 2.94] | 2 | 2.38 | [2.26, 2.50] | 1 |
| [8] Not be able to get it out of your mind. | 2.42 | [2.32, 2.52] | 2 | 2.70 | [2.45, 2.95] | 1 | 3.00 | [2.79, 3.21] | 1 | 3.01 | [2.74, 3.28] | 1 | 2.12 | [2.00, 2.24] | 2 |
| [20] Hold a grudge. | 2.15 | [2.05, 2.25] | 3 | 2.38 | [2.12, 2.64] | 6 | 2.45 | [2.19, 2.71] | 4 | 2.61 | [2.34, 2.88] | 3 | 1.95 | [1.83, 2.07] | 3 |
| [10] Get into an argument. | 2.09 | [1.99, 2.19] | 4 | 2.67 | [2.43, 2.91] | 2 | 2.13 | [1.87, 2.39] | 9 | 2.54 | [2.25, 2.83] | 5 | 1.88 | [1.76, 2.00] | 7 |
| [5] Withdraw into yourself, shut everything out. | 2.03 | [1.93, 2.13] | 5 | 2.17 | [1.89, 2.45] | 9 | 2.54 | [2.27, 2.81] | 3 | 2.57 | [2.26, 2.88] | 4 | 1.79 | [1.66, 1.92] | 9 |
| [13] Do something you find comforting (put headphones in, use a fidget toy, or other sensory activity). | 1.99 | [1.89, 2.09] | 6 | 1.97 | [1.71, 2.23] | 11 | 2.15 | [1.90, 2.40] | 8 | 2.19 | [1.88, 2.50] | 9 | 1.92 | [1.79, 2.05] | 5 |
| [11] Leave the situation to find your own space. | 1.95 | [1.86, 2.04] | 7 | 1.88 | [1.64, 2.12] | 12 | 2.24 | [2.00, 2.48] | 6 | 1.76 | [1.50, 2.02] | 15 | 1.93 | [1.82, 2.04] | 4 |
| [24] Switch or swing from one emotion to another. | 1.88 | [1.78, 1.98] | 8 | 2.56 | [2.31, 2.81] | 5 | 2.33 | [2.08, 2.58] | 5 | 2.46 | [2.16, 2.76] | 6 | 1.53 | [1.42, 1.64] | 14 |
| [18] Look for explanations for why the situation has occurred. | 1.82 | [1.73, 1.91] | 9 | 1.68 | [1.41, 1.95] | 15 | 2.02 | [1.77, 2.27] | 10 | 1.34 | [1.08, 1.60] | 18 | 1.89 | [1.78, 2.00] | 6 |
| [9] Confront the person/situation. | 1.78 | [1.69, 1.87] | 10 | 2.12 | [1.86, 2.38] | 10 | 1.71 | [1.47, 1.95] | 15 | 1.97 | [1.65, 2.29] | 13 | 1.69 | [1.57, 1.81] | 12 |
| [4] Storm off. | 1.76 | [1.66, 1.86] | 11 | 2.64 | [2.37, 2.91] | 3 | 1.88 | [1.63, 2.13] | 11 | 2.41 | [2.08, 2.74] | 8 | 1.43 | [1.32, 1.54] | 17 |
| [1] Shout and/or swear (either or both) | 1.74 | [1.64, 1.84] | 12 | 2.34 | [2.07, 2.61] | 7 | 1.78 | [1.53, 2.03] | 13 | 2.46 | [2.14, 2.78] | 7 | 1.48 | [1.36, 1.60] | 15 |
| [14] Re-think things, not blow things out of proportion. | 1.65 | [1.56, 1.74] | 13 | 1.38 | [1.14, 1.63] | 18 | 1.43 | [1.21, 1.65] | 19 | 1.11 | [0.86, 1.36] | 23 | 1.86 | [1.75, 1.97] | 8 |
| [17] Look for support from others. | 1.65 | [1.56, 1.74] | 14 | 1.47 | [1.23, 1.71] | 17 | 1.87 | [1.65, 2.09] | 12 | 1.38 | [1.11, 1.65] | 16 | 1.69 | [1.58, 1.8] | 13 |
| [16] Distract yourself by thinking about positive things. | 1.58 | [1.49, 1.67] | 15 | 1.29 | [1.05, 1.53] | 20 | 1.48 | [1.25, 1.71] | 17 | 1.13 | [0.86, 1.40] | 22 | 1.74 | [1.63, 1.85] | 11 |
| [22] Feel physically sick (e.g., have a headache, stomach pain). | 1.54 | [1.44, 1.64] | 16 | 1.7 | [1.42, 1.98] | 14 | 2.2 | [1.93, 2.47] | 7 | 2.05 | [1.69, 2.41] | 11 | 1.26 | [1.14, 1.38] | 19 |
| [15] Try not to think about it, and let it pass. | 1.53 | [1.44, 1.62] | 17 | 1.24 | [1.00, 1.48] | 21 | 1.19 | [0.98, 1.40] | 22 | 1.00 | [0.75, 1.25] | 24 | 1.77 | [1.66, 1.88] | 10 |
| [21] Use strategies you’ve ‘learnt’ to manage your feelings (e.g., taking deep breaths, counting down). | 1.44 | [1.35, 1.53] | 18 | 1.33 | [1.08, 1.58] | 19 | 1.64 | [1.40, 1.88] | 16 | 1.23 | [0.95, 1.51] | 21 | 1.46 | [1.35, 1.57] | 16 |
| [12] Talk to an adult. | 1.32 | [1.23, 1.41] | 19 | 1.19 | [0.95, 1.44] | 22 | 1.35 | [1.14, 1.56] | 20 | 1.35 | [1.07, 1.63] | 17 | 1.34 | [1.22, 1.46] | 18 |
| [23] Refuse to do what a school staff member asks you to do. | 1.29 | [1.19, 1.39] | 20 | 2.24 | [1.95, 2.53] | 8 | 1.47 | [1.2, 1.74] | 18 | 2.18 | [1.85, 2.51] | 10 | 0.89 | [0.79, 0.99] | 22 |
| [19] Neglect your daily routine (e.g., stop sleeping or eating well). | 1.23 | [1.14, 1.32] | 21 | 1.48 | [1.21, 1.75] | 16 | 1.77 | [1.51, 2.03] | 14 | 2.03 | [1.72, 2.34] | 12 | 0.92 | [0.81, 1.02] | 21 |
| [2] Hit out/punch objects. | 1.20 | [1.11, 1.29] | 22 | 1.85 | [1.59, 2.11] | 13 | 1.19 | [0.95, 1.43] | 21 | 1.89 | [1.55, 2.23] | 14 | 0.94 | [0.84, 1.04] | 20 |
| [3] Get into a fight. | 0.78 | [0.70, 0.86] | 23 | 1.15 | [0.93, 1.37] | 23 | 0.75 | [0.57, 0.93] | 24 | 1.24 | [0.94, 1.54] | 20 | 0.63 | [0.54, 0.72] | 23 |
| [7] Hurt yourself. | 0.78 | [0.69, 0.86] | 24 | 1.12 | [0.86, 1.38] | 24 | 1.12 | [0.88, 1.36] | 23 | 1.27 | [0.96, 1.58] | 19 | 0.54 | [0.45, 0.63] | 24 |
| (ii) **MERSI Domain Scores** | | | | | | | | | | | | | | | |
|  | M | SD |  | M | SD |  | M | SD |  | M | SD |  | M | SD |  |
| Total ODE score | 10.7 | 6.56 | -- | 15.0 | 6.12 | -- | 10.9 | 6.09 | -- | 14.7 | 7.56 | -- | 8.94 | 5.78 | -- |
| Total IDE score | 11.1 | 5.71 | -- | 12.8 | 4.90 | -- | 13.9 | 5.30 | -- | 14.0 | 5.54 | -- | 9.62 | 5.48 | -- |
| Total SFR score | 11.0 | 5.59 | -- | 9.58 | 5.64 | -- | 11.0 | 4.59 | -- | 8.54 | 5.88 | -- | 11.7 | 5.56 | -- |

Note. The mean, 95% confidence interval, and item rank of the MERSI items (including those items that were eventually rejected) in the overall and each participant group. Rank was generated based on the mean values, where the top rank (the smallest number) represents a higher mean. Abbreviation. ADHD=attention-deficit/hyperactivity disorder, ODE=Outward Distress Expressions, IDE=Inward Distress Expressions, and SFR =Solution-Focused Responses

## Measurement Equivalence

The final model explained 42% of the variance in the data with an acceptable model fit (Likelihood Chi2 = 782, *p* <.001; TLI = 0.814; RMSEA = 0.082, 90% CI [0.076, 0.087]; BIC = -95.7; SRMR = 0.05). In the multigroup CFA, accounting for groups (ADHD, autism, combined, and control), biological sex (male and female), and ethnicity (white and minoritised ethnic group) resulted in reduced model fits in general (see below), but the differences in CFI and RMSEA were minor during the metric invariance model fitting (ΔCFIs ≤ .005, ΔRMSEA≤ .003), where we also saw a favourable reduction of AIC. This suggests that factor loadings contribute to the three-factor model somewhat similarly across groups [8], although the X^2^ model fit remained (unfavourably) significant in some cases. The AIC increased during scalar invariance model fitting, but measurement invariance at a stringent level was not expected for the MERSI (Table S6).

**Table S6. Measurement Invariance of the EB Index Across Various Subgrouping**

| Invariance model | Estimated parameters | *X*^2^ | *df* | *p* | AIC | CFI | RMSEA | SRMR | ΔCFI | ΔRMSEA |
| --- | --- | --- | --- | --- | --- | --- | --- | --- | --- | --- |
| (a) Groups (ADHD, autism, combined, control) | | | | | | | | | | |
| Configural | 252 | 1742.4 | 668 | .011 | 44397 | 0.771 | 0.094 | 0.088 | -- | -- |
| Metric | 252 | 1817.4 | 719 | .016 | 44370 | 0.766 | 0.091 | 0.093 | -0.005 | -0.003 |
| Scalar | 261 | 1987.3 | 770 | <.0001 | 44438 | 0.741 | 0.093 | 0.096 | -0.025 | 0.002 |
| (b) Sex (male, female) | | | | | | | | | | |
| Configural | 126 | 1334.2 | 334 | .452 | 44409 | 0.800 | 0.091 | 0.079 | -- | -- |
| Metric | 126 | 1375.2 | 351 | <.001 | 44402 | 0.795 | 0.089 | 0.084 | -0.005 | -0.002 |
| Scalar | 129 | 1491.4 | 368 | <.0001 | 44491 | 0.775 | 0.091 | 0.086 | -0.020 | 0.002 |
| (c) Ethnicity (white, minoritised ethnic group) | | | | | | | | | | |
| Configural | 126 | 1329.4 | 334 | <.0001 | 44601 | 0.802 | 0.090 | 0.080 | -- | -- |
| Metric | 126 | 1355.6 | 351 | 0.289 | 44594 | 0.800 | 0.088 | 0.083 | -0.002 | -0.002 |
| Scalar | 129 | 1459.6 | 368 | <0.0001 | 44664 | 0.783 | 0.090 | 0.085 | -0.017 | 0.002 |
| Note. Measurement invariance was investigated across (a) Groups (ADHD, autism, combined [ADHD+autism], control), (b) sex (c), and (d) race. Model fit metrics were observed in the configural, metric, and scalar invariance models. Abbreviation. X2=chi square statistics, df = degrees of freedom, p= significance. AIC=Akaike Information Criterion, CFI= Comparative Fit Index, RMSEA=Root Mean Square Error of Approximation, SRMR=Standardized Root Mean Square Residual. | | | | | | | | | | |

## Correlation between MERSI and DERS Domain Scores

The MERSI domain ODE and IDE scores were positively correlated (Pearson’s *r*=0.39, *p*<.001) with a medium effect size, and each was correlated negatively with the SFR domain (*r*=-0.12 and -0.17, *p*s<.01) with a small effect size. MERSI ODE and IDE scores showed positive correlations with DERS domain scores, with effect sizes ranging from ‘small’ to ‘large’ (*r*s_ODE_=0.14-0.53, *p*s<.001; *r*s_IDE_=0.22-0.65, *p*s<.001). The SFR domain score is negatively correlated with the DERS domain scores, with effect sizes ranging from small to medium (*r*s=-0.14- -0.38, *p*s<.001) (Table S7).

**Table S7. Pairwise Correlations Table**

|  | MERSI | | | | DERS | | | | | |
| --- | --- | --- | --- | --- | --- | --- | --- | --- | --- | --- |
|  | ODE | IDE | SFR | Awareness | | Clarity | Goal | Impulse | Nonacceptance | Strategies |
| MERSI - IDE | 0.39*** | -- | -- | -- | | -- | -- | -- | -- | -- |
| MERSI - SFR | -0.17*** | -0.12** | -- | -- | | -- | -- | -- | -- | -- |
| DERS - Awareness | 0.22*** | 0.22*** | -0.38*** | -- | | -- | -- | -- | -- | -- |
| DERS - Clarity | 0.25*** | 0.55*** | -0.26*** | 0.30*** | | -- | -- | -- | -- | -- |
| DERS - Goal | 0.37*** | 0.57*** | -0.21*** | 0.16*** | | 0.52*** | -- | -- | -- | -- |
| DERS - Impulse | 0.53*** | 0.46*** | -0.28*** | 0.26*** | | 0.54*** | 0.61*** | -- | -- | -- |
| DERS - Nonacceptance | 0.14*** | 0.49*** | -0.14*** | 0.10** | | 0.58*** | 0.49*** | 0.44*** | -- | -- |
| DERS - Strategies | 0.26*** | 0.57*** | -0.24*** | 0.14*** | | 0.63*** | 0.63*** | 0.61*** | 0.62*** | -- |
| DERS - Total | 0.41*** | 0.65*** | -0.33*** | 0.43*** | | 0.80*** | 0.79*** | 0.80*** | 0.74*** | 0.83*** |
| Abbreviation. MERSI=My Emotional Response in School Inventory, DERS=Difficulties in Emotion Regulation Scale, ODE=outward distress expression, IDE=inward distress expression, SFR=solution-focused response, colour hue indicates strengths of correlation ( = 1.0;  = -1.0). | | | | | | | | | | |

## The Influences of Formal Diagnosis Status on MERSI Scores

The mean and standard deviation of MERSI ODE, IDE, and SFR scores in the subgroups with and without formal diagnosis within each neurodivergent group are presented in Table S8. The additional full-factorial 2(formal diagnosis status)×3(groups) ANOVA models performed separately on the ODE, IDE and SFR scores, showed a main effect of group on ODE and SFR scores (*F*_ODE_[2, 273]=11.8, *p*<.001, *η*_p_^2^=0.079; *F*_SFR_[2, 273]=4.66, *p*=.010, *η*_p_^2^=0.033), but not on the IDE score (*F*_IDE_[2,273]=1.41, *p*=.25, *η*_p_^2^=0.01).

*Post-hoc* pairwise comparisons, corrected using TukeyHSD, showed that ODE responses were significantly higher in the ADHD group (*ΔEMM=*4.01, *d*=0.61, *p*=.004), and the combined group (*ΔEMM=*4.03, *d*=0.62, *p*<.001) relative to the autistic group. Furthermore, the SFR responses were significantly lower in the combined group compared to the autistic group (*ΔEMM=-*2.68, *d*=-0.50, *p*=.007). These were consistent with the effects found on the responses using a four-level participant group, which included the control participants (see main manuscript Figure 1C).

There was neither a significant main effects of formal diagnosis status on all domains of MERSI responses (*F*_ODE_[1, 273]=2.86, *p*=.09, *η*_p_^2^=0.009; *F*_IDE_[1, 273]= 0.004, *p*=.952, *η*_p_^2^=0.00001; *F*_SFR_[1, 273]=0.55, *p*=.460, *η*_p_^2^=0.002), nor was there a significant interaction between formal diagnosis status × group on these dependent variables (*F*_ODE_[2, 273]=0.25, *p*=.781, *η*_p_^2^=0.002; *F*_IDE_[2, 273]=0.55, *p*=.460, *η*_p_^2^=0.001; *F*_SFR_[2, 273]=0.5, *p*=.521, *η*_p_^2^=0.005), supporting the decision to collapse the subgroups with/without formal diagnosis within the neurodivergent categories, i.e., ADHD, autism, and combined groups.

**Table S8. MERSI Scores across Groups by Formal Diagnosis Status**

|  | ADHD | | | Autism | | | Combined | | | Group differences | | |
| --- | --- | --- | --- | --- | --- | --- | --- | --- | --- | --- | --- | --- |
|  | Dx (n=89)  M (SD) | No dx (n=11)  M (SD) | Dx-No dx  Adj. *d* | Dx (n=65)  M (SD) | No dx (n=35)  M (SD) | Dx-No dx  Adj. *d* | Dx (n=57)  M (SD) | No dx (n=22)  M (SD) | Dx-No dx  Adj. *d* | ADHD-Autism  Adj. *d* | ADHD-Combined Adj. *d* | Autism-Combined Adj. *d* |
| Total ODE score | 15.0 (6.18) | 15.4 (5.85) | -0.06 | 10.3 (6.51) | 12.0 (5.12) | -0.25 | 14.1 (7.40) | 16.3 (7.88) | -0.35 | **0.61**** | -0.003 | **-0.62***** |
| Total IDE score | 12.8 (4.98) | 13.0 (4.38) | -0.03 | 14.4 (5.08) | 12.9 (5.64) | 0.28 | 13.4 (5.72) | 15.3 (4.92) | -0.36 | -0.15 | -0.28 | -0.14 |
| Total SFR score | 9.47 (5.59) | 10.5 (6.22) | -0.18 | 11.2 (4.46) | 10.6 (4.88) | 0.11 | 8.96 (6.09) | 7.45 (5.25) | 0.28 | -0.17 | 0.32 | **0.50**** |

Abbreviation. ADHD=attention-deficit/hyperactivity disorder, dx=diagnosis, ODE=Outward Distress Expressions, IDE=Inward Distress Expressions, and SFR =Solution-Focused Responses, M=mean, SD=standard deviation. Adj. *d*= Adjusted Cohen’s *d*. *** *p*<.001, ***p* <.01

## Adjusted Group Differences of MERSI Responses

MERSI ODE, IDE, and SFR mean scores within participant group, stratified by age, sex (female [M], male [M]) and ethnicity (white [W], minoritised ethnic group [M]) are presented in Table S9. MERSI group differences remained after adjusting for the group differences in age, sex, and ethnicity.

**Table S9. MERSI Domain Scores Stratified by Age, Sex, and Ethnicity**

| MERSI  Domain | Covariate | ADHD | | Autism | | Combined | | Control | |
| --- | --- | --- | --- | --- | --- | --- | --- | --- | --- |
|  |  | n | M (SD) | n | M (SD) | n | M (SD) | n | M (SD) |
| (1) ODE | Age 11 | 6 | 18.0 (8.83) | 7 | 14.0 (5.92) | 6 | 22.3 (3.78) | 13 | 8.42 (5.87) |
|  | Age 12 | 35 | 12.3 (5.72) | 21 | 10.9 (7.23) | 28 | 13.1 (7.10) | 120 | 9.29 (5.80) |
|  | Age 13 | 26 | 15.8 (4.77) | 22 | 12.0 (6.01) | 17 | 16.3 (8.90) | 138 | 8.30 (5.30) |
|  | Age 14 | 17 | 18.4 (5.97) | 19 | 8.58 (6.53) | 12 | 13.1 (6.53) | 88 | 8.70 (6.18) |
|  | Age 15 | 12 | 15.0 (6.02) | 19 | 11.2 (5.08) | 9 | 14.0 (6.40) | 74 | 9.92 (6.29) |
|  | Age 16 | 4 | 14.3 (7.14) | 12 | 10.5 (4.52) | 7 | 14.3 (8.67) | 20 | 8.65 (5.02) |
| (2) IDE | Age 11 | 6 | 10.0 (5.14) | 7 | 15.0 (5.89) | 6 | 13.7 (3.27) | 13 | 11.3 (4.91) |
|  | Age 12 | 35 | 11.8 (4.62) | 21 | 13.9 (5.29) | 28 | 13.9 (6.05) | 120 | 10.1 (4.76) |
|  | Age 13 | 26 | 13.3 (4.69) | 22 | 12.8 (5.84) | 17 | 15.8 (6.19) | 138 | 8.84 (5.68) |
|  | Age 14 | 17 | 13.4 (5.37) | 19 | 13.1 (5.92) | 12 | 13.2 (3.51) | 88 | 9.99 (6.24) |
|  | Age 15 | 12 | 15.6 (4.81) | 19 | 13.5 (4.75) | 9 | 11.8 (4.99) | 74 | 9.64 (5.46) |
|  | Age 16 | 4 | 13.3 (4.65) | 12 | 17.1 (2.91) | 7 | 14.3 (7.04) | 20 | 9.45 (5.00) |
| (3) SFR | Age 11 | 6 | 4.50 (4.85) | 7 | 10.7 (7.23) | 6 | 4.50 (4.85) | 13 | 11.2 (6.07) |
|  | Age 12 | 35 | 9.00 (5.38) | 21 | 10.9 (4.05) | 28 | 9.00 (5.37) | 120 | 12.0 (5.10) |
|  | Age 13 | 26 | 11.5 (6.43) | 22 | 9.73 (5.08) | 17 | 7.65 (5.70) | 138 | 11.7 (5.95 |
|  | Age 14 | 17 | 8.06 (4.99) | 19 | 10.0 (4.23) | 12 | 11.7 (6.18) | 88 | 12.1 (5.48) |
|  | Age 15 | 12 | 8.75 (3.82) | 19 | 12.1 (4.18) | 9 | 7.22 (5.72) | 74 | 10.9 (5.42) |
|  | Age 16 | 4 | 12.3 (4.64) | 12 | 13.3 (3.37) | 7 | 8.71 (7.61) | 20 | 12.5 (6.16) |
| (1) ODE | Sex (F) | 29 | 14.6 (6.86) | 56 | 11.0 (6.63) | 31 | 14.4 (7.59) | 148 | 8.20 (5.53) |
|  | Sex (M) | 71 | 15.2 (5.83) | 44 | 10.8 (5.39) | 48 | 14.9 (7.61) | 304 | 9.33 (5.86) |
| (2) IDE | Sex (F) | 29 | 15.2 (5.08) | 56 | 15.1 (4.58) | 31 | 16.8 (4.01) | 148 | 12.3 (5.42) |
|  | Sex (M) | 71 | 11.9 (4.52) | 44 | 12.4 (5.80) | 48 | 12.1 (5.65) | 304 | 8.33 (5.00) |
| (3) SFR | Sex (F) | 29 | 8.14 (4.91) | 56 | 10.9 (4.68) | 31 | 8.55 (5.89) | 148 | 12.2 (4.75) |
|  | Sex (M) | 71 | 10.2 (5.84) | 44 | 11.1 (4.53) | 48 | 8.54 (5.93) | 304 | 11.5 (5.91) |
| (1) ODE | Ethnicity (W) | 77 | 14.6 (6.46) | 64 | 10.7 (5.63) | 56 | 15.0 (7.05) | 187 | 8.70 (5.71) |
|  | Ethnicity (M) | 23 | 16.2 (4.70) | 36 | 11.3 (6.91) | 23 | 13.9 (8.79) | 266 | 9.11 (5.84) |
| (2) IDE | Ethnicity (W) | 77 | 13.2 (4.63) | 64 | 14.9 (4.53) | 56 | 14.1 (5.54) | 187 | 10.2 (5.71) |
|  | Ethnicity (M) | 23 | 11.6 (5.64) | 36 | 12.2 (6.14) | 23 | 13.7 (5.65) | 266 | 9.22 (5.29) |
| (3) SFR | Ethnicity (W) | 77 | 9.40 (5.07) | 64 | 10.8 (4.19) | 56 | 8.09 (5.44) | 187 | 11.9 (5.00) |
|  | Ethnicity (M) | 23 | 10.2 (7.33) | 36 | 11.2 (5.29) | 23 | 9.65 (6.83) | 266 | 11.7 (5.92) |

Abbreviations. ADHD=attention-deficit/hyperactivity disorder, n= number of participants, M=mean, SD=standard deviation, ODE=Outward Distress Expressions, IDE=Inward Distress Expressions, and SFR=Solution-Focused Responses. F=female, M=male, W=white, and M=minoritised ethnic group.

Group differences remained after adjusting for age, sex, and ethnicity for the MERSI ODE (*F* [3, 715]=40.4, *p*<.001, *η*_p_^2^=0.14), IDE (*F*[3, 715]=35.2, *p*<.001, *η*_p_^2^=0.11), and SFR (*F* [3, 715]=10.3, *p*<.001, *η*_p_^2^=0.04)(Table S10). The ODE were higher in all neurodivergent groups relative to the control group (ADHD: *ΔEMM*=6.83, *d*=1.12, *p*<.001; autism: *ΔEMM*=2.32, *d*=0.38, *p*=.007; combined: *ΔEMM*=5.59, *d*=0.92, *p*<.001). Higher ODE was also observed in the ADHD and combined groups relative to the autistic group (ADHD: *ΔEMM*= 4.51, *d*=0.74, *p*<.001; combined: *ΔEMM*=3.27, *d*=0.54, *p*=.006).

The IDE was higher in all three neurodivergent groups (ADHD: *ΔEMM*=3.15, *d*=0.62; autism: *ΔEMM*=2.97, *d*=0.59; combined: *ΔEMM*=3.94, *d*=0.78, all *p*s<.001) relative to the control groups and did not differ among the neurodivergent groups. There were additional main effects of sex (*F*[1, 715]=93.1, *p*<.001, *η*_p_^2^=0.10) and ethnicity (*F*[1, 715]=4.68, *p*<.031, *η*_p_^2^=0.005) found for the IDE. Higher IDE was demonstrated by females than males (*ΔEMM*=3.72, *d*=0.74, *p*<.001) and white than minoritised ethnic group (*ΔEMM*=0.86, *d*=0.17, *p*=0.031).

Finally, the SFR was lower in the groups with ADHD compared to the control group only (ADHD: *ΔEMM*=-2.54, *d*=-0.46, *p*=.008; combined: *ΔEMM*=-2.97, *d*=-0.53, *p*<.001)

**Table S10. Group Effects on MERSI Domain Adjusted by Age, Sex and Ethnicity**

| Effects | ODE | | | | IDE | | | | SFR | | | |
| --- | --- | --- | --- | --- | --- | --- | --- | --- | --- | --- | --- | --- |
|  | df | *F* | *p* | *η*_p_^2^ | df | *F* | *P* | *η*_p_^2^ | Df | *F* | *p* | *η*_p_^2^ |
| Main effects |  |  |  |  |  |  |  |  |  |  |  |  |
| ***Group*** | **3, 715** | **40.4** | **<.001***** | **0.14** | **3, 715** | **35.2** | **<.001***** | **0.11** | **3, 715** | **10.3** | **<.001***** | **0.04** |
| *Age* | 1, 715 | 0.02 | .888 | 0.00002 | 1, 715 | 0.32 | .573 | 0.0003 | 1, 715 | 0.53 | 0.468 | 0.0007 |
| *Sex* | 1, 715 | 2.59 | .108 | 0.003 | 1, 715 | 93.1 | <.001 | 0.10 | 1, 715 | 0.12 | 0.725 | 0.0002 |
| *Ethnicity* | 1, 715 | 0.58 | .446 | 0.0007 | 1, 715 | 4.68 | .031 | 0.005 | 1, 715 | 0.10 | 0.748 | 0.0001 |
| Interactions |  |  |  |  |  |  |  |  |  |  |  |  |
| *Group × Age* | 3, 715 | 1.31 | .269 | 0.005 | 3, 715 | 1.79 | .147 | 0.006 | 3, 715 | 0.83 | 0.478 | 0.003 |
| *Group × Sex* | 3, 715 | 0.45 | .720 | 0.002 | 3, 715 | 0.72 | .539 | 0.002 | 3, 715 | 1.47 | 0.222 | 0.006 |
| *Group × Ethnicity* | 3, 715 | 0.56 | .642 | 0.002 | 3, 715 | 1.23 | .297 | 0.004 | 3, 715 | 0.44 | 0.722 | 0.002 |

Abbreviations. ODE=Outward Distress Expressions, IDE=Inward Distress Expressions, and SFR=Solution-Focused Responses, *df*=degrees of freedom. ****p*<.001

## Adjusted Cross-sectional Prediction of Depression or Anxiety Using the MERSI Domain Scores

The adjusted association between MERSI domain scores, EB and ERD with depression or anxiety are presented in Table S11.

**Table S11. Specificity of Association between MERSI Domain Scores and Depression or Anxiety**

| Predictors | Depression | | | | Anxiety | | | | |
| --- | --- | --- | --- | --- | --- | --- | --- | --- | --- |
|  | *b* | *95%*CI | *t-*value | *p* | *b* | *95%CI* | *t-*value | *p* |  |
| EB | **0.19** | **[0.11, 0.26]** | **5.07** | **<.001***** | **0.24** | **[0.18, 0.31]** | **7.15** | **<.001***** |  |
| ODE | -0.04 | [-0.10, 0.02] | -1.34 | .180 | **-0.11** | **[-0.16, -0.05]** | **-3.93** | **<.001***** |  |
| IDE | **0.24** | **[0.16, 0.32]** | **6.19** | **<.001***** | **0.30** | **[0.23, 0.37]** | **8.25** | **<.001***** |  |
| SFR | 0.03 | [-0.02, 0.08] | 1.04 | .298 | 0.01 | [-0.04, 0.06] | 0.44 | .664 |  |
| ERD | **0.34** | **[0.25, 0.43]** | **7.74** | **<.001***** | **0.31** | **[0.23, 0.39]** | **7.48** | **<.001***** |  |
| Alexithymia | **0.13** | **[0.05, 0.21]** | **3.12** | **.002**** | **0.11** | **[0.04, 0.19]** | **2.97** | **.003**** |  |
| Age | **0.08** | **[0.03, 0.12]** | **3.07** | **.002**** | **0.05** | **[0.00, 0.09]** | **2.07** | **.039*** |  |
| Sex | -0.07 | [-0.18, 0.04] | -1.23 | .220 | -0.02 | [-0.12, 0.08] | -0.37 | .713 |  |
| Ethnicity | 0.10 | [-0.00, 0.20] | 1.93 | .054 | 0.02 | [-0.07, 0.11] | 0.47 | .638 |  |

Abbreviations. *b*=standardised beta, 95%CI=95% confidence interval, EB=emotional burden, ODE=outward distress expression, IDE=inward distress expression, SFR=solution-focused responses, ERD=emotional regulation deficit. ****p*<.001, ***p*<.01, **p*<.05

## Group Differences in the Adjusted Cross-sectional Prediction of Mental Health Problems

Table S12 summarises analyses examining the adjusted cross-sectional prediction of depression or anxiety using MERSI domain scores.

**Table S12. Modification of the Adjusted Association Between MERSI Domain Scores with Depression or Anxiety by Group**

| Predictors | Depression | | | | Anxiety | | | |
| --- | --- | --- | --- | --- | --- | --- | --- | --- |
|  | *b* | *95%*CI | *t-*value | *p* | *b* | *95%*CI | *t-*value | *p* |
| **(i) ODE** | | | | | | | | |
| MERSI – ODE | **0.13** | **[0.05, 0.21]** | **3.17** | **.002**** | **0.12** | **[0.04, 0.20]** | **2.90** | **.004**** |
| Group – ADHD | 0.15 | [-0.05, 0.36] | 1.48 | .141 | 0.15 | [-0.04, 0.36] | 1.55 | .122 |
| Group – Autism | **0.20** | **[0.03, 0.38]** | **2.31** | **.021*** | **0.30** | **[0.11, 0.45]** | **3.28** | **.001**** |
| Group – Combined | 0.17 | [-0.04, 0.37] | 1.57 | .118 | **0.21** | **[0.01, 0.42]** | **2.04** | **.042*** |
| Alexithymia | **0.54** | **[0.48, 0.60]** | **16.9** | **<.001***** | **0.55** | **[0.49, 0.61]** | **17.5** | **<.001***** |
| Age | **0.09** | **[0.04, 0.15]** | **-4.89** | **<.001***** | **0.06** | **[0.01, 0.12]** | **2.23** | **.026*** |
| Sex | **-0.30** | **[-0.42, -0.18]** | **3.35** | **<.001***** | **-0.30** | **[-0.42, -0.18]** | **-5.03** | **<.001***** |
| Ethnicity | 0.05 | [-0.07, 0.17] | 0.86 | .390 | -0.03 | [-0.14, 0.09] | -0.48 | .632 |
| ODE × Group – ADHD | -0.01 | [-0.18, 0.17] | -0.07 | .947 | -0.02 | [-0.19, 0.16] | -0.22 | .826 |
| ODE × Group – Autism | 0.04 | [-0.14, 0.22] | 0.46 | .647 | -0.16 | [-0.34, 0.01] | -1.81 | .071 |
| ODE × Group – Combined | -0.08 | [-0.25, 0.08] | -0.97 | .335 | -0.10 | [-0.26, 0.07] | -1.17 | .241 |
| **(ii) IDE** | | | | | | | | |
| MERSI – IDE | **0.40** | **[0.32, 0.48]** | **9.70** | **<.001***** | **0.42** | **[0.34, 0.49]** | **10.8** | **<.001***** |
| Group – ADHD | **0.20** | **[0.04, 0.37]** | **2.40** | **.017*** | **0.17** | **[0.02, 0.33]** | **2.15** | **.032*** |
| Group – Autism | 0.12 | [-0.05, 0.30] | 1.41 | .158 | 0.16 | [-0.01, 0.32] | 1.89 | .060 |
| Group – Combined | 0.08 | [-0.11, 0.28] | 0.84 | .400 | 0.11 | [-0.08, 0.29] | 1.15 | .249 |
| Alexithymia | **0.35** | **[0.29, 0.42]** | **10.3** | **<.001***** | **0.33** | **[0.26, 0.39]** | **10.1** | **<.001***** |
| Age | **0.09** | **[0.04, 0.14]** | **3.31** | **<.001***** | **0.05** | **[0.00, 0.10]** | **2.16** | **.031*** |
| Sex | -0.09 | [-0.21, 0.02] | -1.60 | .111 | -0.08 | [-0.19, 0.03] | -1.45 | .147 |
| Ethnicity | **0.12** | **[0.01, 0.22]** | **2.10** | **.036*** | 0.04 | [-0.06, 0.15] | 0.82 | .413 |
| IDE × Group – ADHD | -0.06 | [-0.24, 0.11] | -0.73 | .468 | 0.03 | [-0.14, 0.19] | 0.31 | .754 |
| IDE × Group – Autism | 0.00 | [-0.16, 0.17] | 0.05 | .964 | 0.09 | [-0.07, 0.25] | 1.15 | .251 |
| IDE × Group – Combined | 0.10 | [-0.07, 0.28] | 1.18 | .239 | 0.15 | [-0.02, 0.31] | 1.73 | .084 |
| **(iii) SFR** | | | | | | | | |
| MERSI – SFR | 0.04 | [-0.04, 0.11] | 0.97 | .330 | 0.06 | [-0.01, 0.13] | 1.57 | .116 |
| Group – ADHD | **0.24** | **[0.06, 0.41]** | **2.59** | **.01**** | **0.22** | **[0.05, 0.39]** | **2.48** | **.013*** |
| Group – Autism | **0.24** | **[0.07, 0.41]** | **2.70** | **.007**** | **0.32** | **[0.15, 0.49]** | **3.66** | **<.001***** |
| Group – Combined | 0.20 | [-0.00, 0.40] | 1.95 | .051 | 0.20 | [-0.00, 0.39] | 1.94 | .053 |
| Alexithymia | **0.57** | **[0.51, 0.63]** | **17.8** | **<.001***** | **0.56** | **[0.50, 0.63]** | **18.0** | **<.001***** |
| Age | **0.09** | **[0.04, 0.15]** | **3.29** | **.001**** | **0.07** | **[0.01, 0.12]** | **2.44** | **.015*** |
| Sex | **-0.27** | **[-0.39, -0.15]** | **-4.34** | **<.001***** | **-0.27** | **[-0.39, -0.15]** | **-4.52** | **<.001***** |
| Ethnicity | 0.06 | [-0.06, 0.18] | 1.03 | .303 | -0.02 | [-0.13, 0.10] | -0.29 | .772 |
| SFR × Group – ADHD | -0.12 | [-0.28, 0.05] | -1.38 | .167 | **-0.20** | **[-0.36, -0.03]** | **-2.39** | **.017*** |
| SFR × Group – Autism | -0.01 | [-0.20, 0.19] | -0.08 | .934 | -0.08 | [-0.27, 0.11] | -0.84 | .402 |
| SFR × Group – Combined | -0.05 | [-0.23, 0.12] | -0.60 | .550 | **-0.19** | **[-0.36, -0.02]** | **-2.17** | **.030*** |

Abbreviations. *b*=standardised beta, 95%CI=95% confidence interval, ADHD=attention-deficit/hyperactivity. MERSI=My Emotional Response in School Inventory, ODE=outward distress expression, IDE=inward distress expression, SFR=solution-focused responses, ERD=emotional regulation deficit. ****p*<.001, ***p*<.01, **p*<.05.

# REFERENCES

1.	Streiner DL, Norman GR (2008) Health Measurement Scales : A practical guide to their development and use. Oxford University Press, Incorporated

2.	Cattell RB (1966) The Scree Test For The Number Of Factors. Multivariate Behavioral Research 1:245-276

3.	Horn JL (1965) A rationale and test for the number of factors in factor analysis. Psychometrika 30:179-185

4.	Costello AB, Osborne JW (2005) Best practices in exploratory factor analysis: four recommendations for getting the most from your analysis. Practical Assessment, Research and Evaluation 10:1-9

5.	Bentler PM, Bonett DG (1980) Significance tests and goodness of fit in the analysis of covariance structures. Psychological bulletin 88:588

6.	Gideon S (1978) Estimating the Dimension of a Model. The Annals of Statistics 6:461-464

7.	Davison A, Hinkley D (1997) Bootstrap Methods and Their Application. Journal of the American Statistical Association 94

8.	Chen FF (2007) Sensitivity of Goodness of Fit Indexes to Lack of Measurement Invariance. Structural Equation Modeling: A Multidisciplinary Journal 14:464-504

9.	Overholser BR, Sowinski KM (2008) Biostatistics Primer: Part 2. Nutrition in Clinical Practice 23:76-84

10.	Cohen J (1988) Statistical Power Analysis for the Behavioral Sciences (2nd ed.). Lawrence Erlbaum., Hillsdale, NJ

11.	Lukito S, Chandler S, Kakoulidou M, Griffiths K, Wyatt A, Funnell E, Pavlopoulou G, Baker S, Stahl D, Sonuga-Barke E, RE-STAR team (2025) Emotional burden in school as a source of mental health problems associated with ADHD and/or autism: Development and validation of a new co-produced self-report measure. J Child Psychol Psychiatry, 66(10):1577-1592.
